# A Bottom-Up Coarse-Grained Model for Nucleosome–Nucleosome Interactions with Explicit Ions

**DOI:** 10.1021/acs.jctc.2c00083

**Published:** 2022-05-17

**Authors:** Tiedong Sun, Vishal Minhas, Alexander Mirzoev, Nikolay Korolev, Alexander P. Lyubartsev, Lars Nordenskiöld

**Affiliations:** † School of Biological Sciences, Nanyang Technological University, Singapore 639798; ‡ Department of Materials and Environmental Chemistry, Stockholm University, Stockholm 10691, Sweden

## Abstract

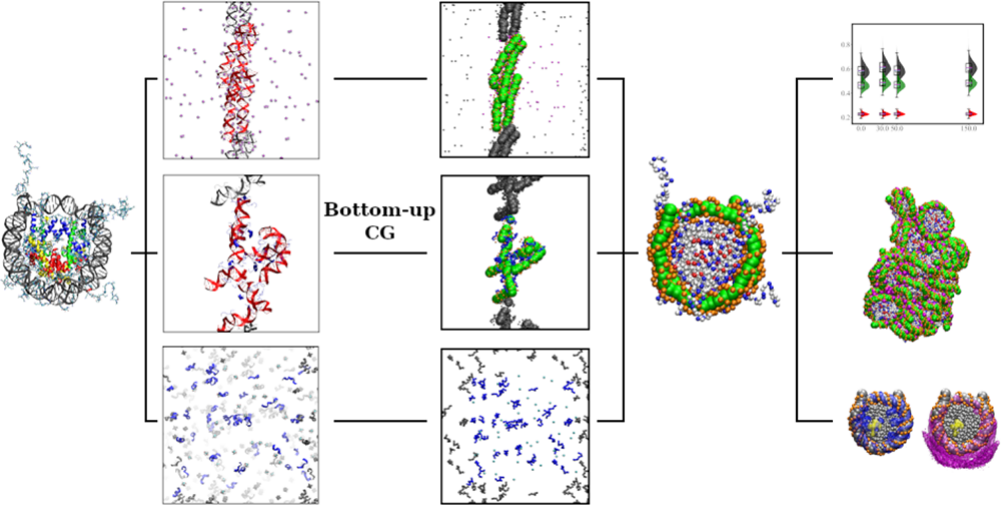

The nucleosome core particle (NCP) is a large complex of 145–147 base pairs of DNA and eight histone proteins and is the basic building block of chromatin that forms the chromosomes. Here, we develop a coarse-grained (CG) model of the NCP derived through a systematic bottom-up approach based on underlying all-atom MD simulations to compute the necessary CG interactions. The model produces excellent agreement with known structural features of the NCP and gives a realistic description of the nucleosome–nucleosome attraction in the presence of multivalent cations (Mg(H_2_O)_6_^2+^ or Co(NH_3_)_6_^3+^) for systems comprising 20 NCPs. The results of the simulations reveal structural details of the NCP–NCP interactions unavailable from experimental approaches, and this model opens the prospect for the rigorous modeling of chromatin fibers.

## 1 Introduction

Eukaryotic cells confine long genomic DNA (about 2 meters in humans) in a small micrometer-sized nucleus through a hierarchy of DNA compaction. The nucleosome, formed by histone proteins and double-stranded DNA, is the first level of eukaryotic DNA compaction. Its central part, the nucleosome core particle (NCP), is a wedge-shaped complex comprised of 145–147 base pairs (bp) of DNA left-handedly wrapped around a histone octamer (HO) consisting of two copies of the histone proteins H2A, H2B, H3, and H4.^1^ Each core histone has a “histone tail,” an unstructured, positively charged N-terminal domain; histone H2A also has a positively charged tail at its C terminus.^2^ The tails play crucial roles in NCP–NCP interaction and the organization of higher-level structures. NCPs are connected by linker DNA of variable length, forming nucleosome arrays and higher-level chromatin structures. The higher-level structures of chromatin, such as the “30 nm fiber,” are less known. Numerous models have been proposed and constantly refined to describe chromatin structure beyond nucleosome arrays.^3^ There is also a continuous debate about the relevance of the in vitro and in silico generated models to the actual structural and dynamic features of the folded chromatin in vivo.^3−5^ However, it is essential to comprehend that a myriad of in vivo and in vitro structures of packed chromatin are possible due to the primary attractive NCP–NCP interaction with secondary contributions from the other nuclear proteins such as linker histones, transcription factors, topoisomerases, chromatin remodeling complexes, etc.

A number of experimental in vitro studies have been conducted on isolated NCPs to understand the organization of NCPs in chromatin.^6−11^ It has been established that histone tails mediate NCP–NCP interaction in a salt-dependent manner, highlighting the polyelectrolyte nature of DNA and the histone proteins.^10^ Furthermore, studies have elucidated different structural organizations of NCPs in condensed phases, the phase diagrams of NCP aggregation, and details of NCP–NCP interactions induced by the presence of multivalent cations (such as Mg^2+^) and high salt.^10,12,13^ It was found that NCP aggregation depends on the charge and nature of the cation.^10,11^ Even though the importance of electrostatic interactions in the structure and dynamics of chromatin is recognized, our understanding of the detailed mechanism is still relatively vague, often depicted by classical polymer theory and highly simplified electrolyte models, such as the Debye–Hückel theory. Additionally, our knowledge of the roles of histone tails in NCP–NCP interaction and chromatin structure was mainly obtained from the effects of mutated and chemically modified histones. The histone tails, highly dynamic, are not resolvable with most imaging techniques.^14^ Hence, the interaction details involving histone tails are hidden from direct experimental observation.

Since as early as the 1990s,^15,16^ numerous modeling and simulation efforts have been made to address the features unattainable through experiments (mentioned above). In early works, individual NCPs in chromatin are modeled as simple solid spheres^16−19^ or ellipsoids.^20,21^ Spherocylinder approximation^22,23^ for the NCP shape and volume became the preferred model after the high-resolution NCP structure was determined.^24,25^ These simple models (for instance, the “two-angle” model^15,19^) are useful in studying the chromatin structure with a few degrees of freedom. However, the significant role of histone tails in NCP–NCP interaction has often been neglected or only crudely assessed. Additionally, detailed interaction features such as the presence of the acidic patch on the HO core surface were omitted or replaced by featureless uniform interactions. Higher-resolution models, such as the explicit tail model,^26^ were developed to mitigate the former issue. An NCP model with refined electrostatic features and a detailed molecular surface has been developed in the Schlick group,^27−30^ which addresses the latter deficiency in NCP modeling. Nevertheless, these models cannot describe all physical interactions mediated by multivalent ions and cellular components. In recent years, a few residue-based coarse-grained (CG) NCP models^31−36^ have been developed as computational capability advances. These models usually adopt bonds to maintain the NCP structure and simple interaction terms, such as Lennard–Jones interactions with mostly empirical top-down derived parameters, to account for the nonbonded interactions. At such level, not only did structural changes accompanied slow dynamic processes, such as DNA unwrapping,^35,36^ but also NCP–NCP interactions due to ion mediated attraction^31^ can be described by the models.

In this paper, we present a newly developed, residue-based CG NCP model derived through systematic bottom-up coarse-graining based on the inverse Monte Carlo (IMC) approach.^37^ The IMC and related approaches such as iterative Boltzmann inversion (IBI)^38^ are referred to as structure-based coarse-graining. In such bottom-up coarse-graining, no empirical parameters are introduced other than the existing all-atom force field parameters of the reference system. We adopt the all-atom CHARMM27 force field^39^ to build our primary all-atom MD simulation systems. The coarse-grained NCP model is parameterized by the IMC method,^37^ in which structural features of the reference model, manifested by the CG particle pair radial distribution functions (RDFs) and distributions of intramolecular bonds and angles, are used to determine interaction potentials of the CG model so that the CG model reproduces these structural features. The resulting NCP model is then validated by comparison with data from in vitro NCP experiments.^38^

A complicating circumstance for systematic bottom-up coarse-graining of NCP is that the NCP is very large for representative atomistic simulations. This makes it very difficult, if not impossible, to run one-step coarse-graining using all-atom MD simulations of the NCP to generate converged structural properties for all interacting species (DNA, protein, and ions). To compute the necessary CG interactions, we overcame this difficulty by performing multiple all-atom MD simulations, each containing a subset of the interacting molecules under the same thermodynamic conditions. Subsequently, the complete CG NCP model is pieced together by inheriting interaction potentials from these subsystems, following the testing of good transferability under the same thermodynamic conditions. We show that our new CG model reproduces well-known structural features of the NCP. Furthermore, the new CG NCP model produces a realistic representation of NCP aggregation induced by multivalent cations. To the best of our knowledge, this work is the first application of a physically rigorous bottom-up coarse-graining to the NCP, a macromolecular complex, and ionic interactions. Additionally, this work is a stepping stone for future physics-based chromatin models combined with an appropriate linker DNA model. We expect such chromatin models based on physics-based interactions to reveal details in chromatin structure, which provide important new insights into chromatin biological functioning.

## 2 Methods

### 2.1 The NCP Model

**Figure 1:**
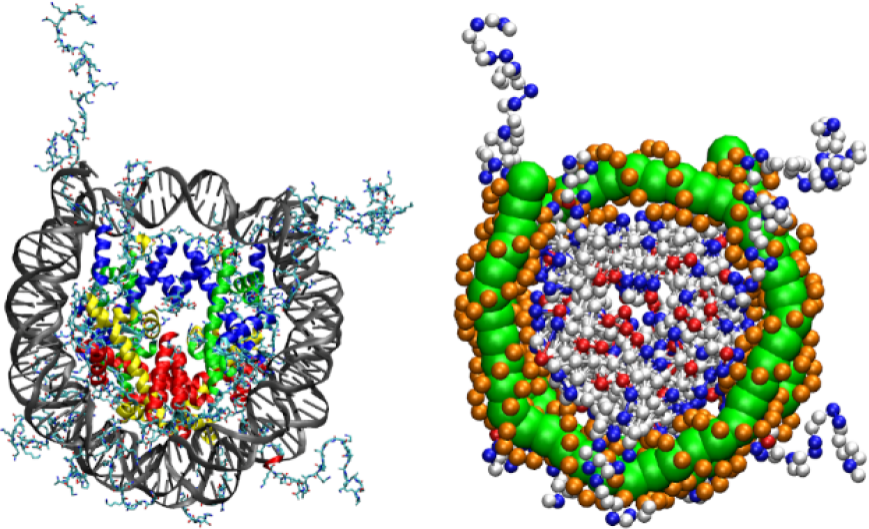
All-atom representation (left) and CG representation (right) of the NCP. The all-atom representation is built using the NCP crystal structure (PDB: 1KX5),^25^ where the globular domains of histone proteins are in colors; DNA is colored gray. The CG NCP structure is shown with positively and negatively charged amino acids colored in blue and red, respectively. Uncharged amino acid beads are displayed as white spheres. The beads representing the phosphate groups in DNA are in orange, while the central DNA beads are shown as green balls.

Coarse-graining of biomolecular systems is always a compromise between the model’s accuracy and computational efficiency. We aim to develop a CG NCP model that contains details of the most important interactions applicable for simulations of a few tens of NCPs to model NCP phase separation to an ordered aggregated state in the presence of multivalent cations, which can later be developed into a chromatin model. We choose the resolution of one bead per amino acid for protein and five beads for every two base pairs of DNA, as depicted in Figure 1, using bottom-up coarse-graining to compute all interaction terms. The nucleosomal DNA is coarse-grained using our previously validated DNA model,^40,41^ where five beads represent every two base pairs. Four of the five beads represent the phosphate group bearing −1e charge. All other atoms in these two base pairs are represented by the fifth bead, located at the center of mass (COM) of these atoms. Ions are modeled explicitly, with one bead representing one ion for all ion species.

The histone proteins are coarse-grained with a resolution of one CG site per one amino acid residue. All amino acids of the histone proteins are represented by beads located at the center of mass (COM) of each amino acid. To simplify the determination and subsequent use of the CG potentials, the 20 natural amino acids are grouped into five bead types corresponding to polar (POL), nonpolar (NPL), positively charged (POS), negatively charged (NEG), and glycine (GLY) amino acids. POS and NEG beads bear charges +e and −e, respectively. Glycine is assigned to a dedicated type because of its small size and higher degrees of backbone freedom. The assignment of amino acid types into CG bead types is listed in Table S1. The same potential functions describe interactions of amino acids belonging to the same type and with other components of the system (DNA and ions). We note that compromise has been made in the bead type definition to balance performance and accuracy. For instance, it has been recently found that even similar residues, such as lysine and arginine, can establish distinct interactions under certain conditions and further affect macroscopic phenomena.^42^ We have conducted a test where lysine and arginine are mapped to two respective CG types. The resulting CG nonbonded potentials (Figure S1) are similar, justifying our bead type definition. This CG bead type definition allows the distinction of essential interactions, including electrostatic, hydrophobic, and hydrophilic, while keeping a minimum number of bead types, avoiding failure in the coarse-graining practice due to the high complexity of the CG model.

The total potential energy defining our NCP model contains four terms:
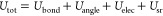
1

The bonded and angular interactions *U*_bond_, *U*_angle_ for DNA, and histone tails are given in a tabulated form, with linear interpolation of potential energy between data points. No angle interaction term is included within the histone core region as it is modeled as an elastic network (discussed below). Angle types are determined by examining X-AA-X angles distributions (“X” stands for any amino acid) for all amino acid types (AA) in the DNA-peptide all-atom simulation system (discussed below). It was found that the angle at proline presents distinctive X-AA-X angle distributions compared with other amino acids. The angle at glycine shows a peak between 120 and 140 degrees instead of a plateau shown by other amino acids (Figure S2). In the end, three angle types along the peptide chain are defined for X-AA-X angles, when AA is proline (denoted Angle_PRO), glycine (Angle_GLY), or other amino acid residues (Angle_Other).

The nonbonded interaction is comprised of electrostatic interaction, *U*_elec_, and short-range nonbonded interaction, *U*_sr_. Electrostatic interactions are determined by integer charges placed on charged amino acids, phosphate groups of the DNA backbone, and explicit ions. The electrostatic interaction follows Coulomb’s law with a constant relative permittivity, ϵ = 78 (eq 2).
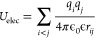
2where *q_i_* and *q_j_* are charges on bead *i* and *j* and *r_ij_* denotes the distance between beads *i* and *j*. The long-range electrostatic interactions are treated by the Particle Mesh Ewald (PME)^43^ or the Particle–Particle Particle–Mesh (PPPM)^44^ methods.

The short-range nonbonded interactions, *U*_sr_, are represented by tabulated pair potentials. Together with bonded potentials, these potentials are generated by the IMC calculation using reference RDFs and distributions of bonds and angles computed in atomistic simulations. For 1–2 and 1–3 bound neighbors, both electrostatic and short-range nonbonded interactions are excluded, whereas the 1–4 and longer interactions are preserved and described in the same way as nonbonded interactions between CG sites of the corresponding types.

We take additional considerations for the wedge shape of the histone core and the highly charged nature of NCP, which are paramount in reproducing the structures of NCP assembly, nucleosome arrays, and chromatin.^45^ Although the nonbonded interactions are parametrized from the atomistic model by structure-based coarse-graining, the histone core structure is not guaranteed to be preserved due to inherent limitations of the underlying all-atom MD simulations. Hence, we use an elastic network model for the histone core since its conformation is much less flexible than the other NCP domains and structurally well-defined. The definition of histone tail regions and core regions is listed in Table S2. The elastic network is built based on bead distances in the experimentally available crystal structure. Any pair of beads with a distance less than 7 Å is connected by a harmonic bond with equilibrium length inherited from the NCP crystal structure:
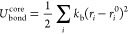
3where a uniform bond strength across all core bonds, *k*_b_, is set to 5 k_B_T/Å^2^. Testing other *k*_b_ values did not produce noticeable changes.

For short-range nonbonded interactions, the core–core terms are treated as excluded volume interactions presented as a repulsive part of the Lennard–Jones potential:
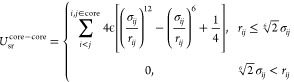
4in which σ_*ij*_ = (σ_*i*_ + σ_*j*_)/2. σ_*ij*_ is determined by the effective radius of each bead type listed in Table S3. ϵ is set to 1.0 k_B_T.

### 2.2 Reference All-Atom MD Simulations

The nucleosome is a large DNA–protein complex, which is difficult to sample directly with the all-atom representation to get reliable and converged structural distributions necessary for bottom-up coarse-graining. Furthermore, the CG NCP model contains many different types of CG sites, which creates difficulties in the bottom-up coarse-graining. We, therefore, implement a “divide and conquer” strategy in which we set up several subsystems, each of which is simpler than a whole NCP. However, taken together, these systems cover all the interactions of the NCP with the ionic environment. Thus, we set up several all-atom MD simulations with subsets of components that will form our CG NCP model. To build a model that is capable of thoroughly investigating the structure and dynamics of NCP and NCP–NCP interaction at the CG level, we aimed to obtain a complete set of interaction potentials for DNA, histone tails, monovalent ions (K^+^, Na^+^, and Cl^–^), and multivalent cations, including hydrated magnesium (Mg(H_2_O)_6_^2+^) and cobalt(III)-hexammine (CoHex^3+^). Among these components, distributions of monovalent ions are usually easy to evaluate. Hence, monovalent ions are present in all reference all-atom subsystems. As all our nonbonded interaction terms are pairwise, we can cover all interactions terms among the three other major components (DNA, histone tails, and multivalent ions) with three simulation setups under the same thermodynamic conditions, each containing a pair of the components, namely DNA-multivalent ions, DNA-peptide, and peptide-multivalent ions.

The setup of all all-atom simulations is summarized in Table S4. This study uses the CHARMM27 force field^39^ to provide reference structural information necessary for the bottom-up coarse-graining. It was shown previously that the CHARMM27 force field provides a better and more stable DNA structure than later updated CHARMM36.^41^ We emphasize that the current CG NCP model is pairwise additive as the underlying all-atom model. Higher-order interactions, such as the multibody effect, are not included, which would not be a problem for models at such a scale. Below, we describe the atomistic simulations of each subsystem separately.

#### 2.2.1 DNA-Multivalent Cation Simulations

In our previous work^41,46^ focused on DNA-multivalent cation interactions, we parameterized a well-performing CG DNA model using IMC. Briefly, the all-atom reference simulations of DNA-multivalent cation systems were set up with four 36 bp long DNA double helices, whose sequences can be found in Table S5. Either Mg(H_2_O)_6_^2+^ or CoHex^3+^ ions were added to reach a total concentration of about 50 mM. It should be noted that upon NCP aggregation and formation of the aggregated concentrated NCP bundles, positive multivalent salt ions are enriched in the NCP phase. Hence, the mean volume average Mg^2+^ ion concentration in the simulation cell (50 mM) corresponds to a significantly lower experimental bulk concentration.^47^

The optimized hydrated Mg(H_2_O)_6_^2+^ model^48^ and optimized CoHex^3+^ model^40^ were used for multivalent ions. About 50 mM K^+^ and 35 mM Na^+^ ions were added to provide a background salt environment close to physiological conditions. Equal amounts of K^+^ and Na^+^ were added in the case of the Mg(H_2_O)_6_^2+^ simulation to neutralize the charge difference between these two systems. We note that although other models of multivalent cations are available, such as the Mg^2+^ model by the Schwierz group,^49^ the multivalent cation models used in the current study are well tested with double-helical DNA. They have been proven to reliably reproduce solution structures.^40,48^ Three independent simulations were performed for the subsystem with CoHex^3+^ ions, having different initial positions and velocities. The CG potentials between all pairs of CG sites were determined by the IMC method. We continue to use this model in the current CG NCP modeling.

#### 2.2.2 DNA-Peptide Simulation

In order to extract interaction potentials between DNA and amino acids, we computed the RDFs between CG sites of DNA and amino acids with all-atom simulations of DNA-peptide systems. Based on the sequence of histone tails, we designed 48 peptides, each 8 amino acids long, containing all combinations of tripeptides found in the histone tails. The sequences of the simulated peptides can be found in Table S6.

The simulation box was built with eight double-helical DNA molecules of 16 bp each and one copy of each of the 48 designed peptides. Sequences of the DNA double helices are listed in Table S7. Although there is one copy of each peptide in the simulation, the relevant degrees of freedom that are subjected to sampling are represented by multiple molecules of peptide (Table S7). Hence, the sampling is not hindered by the number of a specific peptide. About 65 mM K^+^ and 130 mM Na^+^ ions with the neutralizing amount of Cl^–^ ions were added to the system. The cubic simulation box size was approximately 15.1 nm in each dimension. Ten independent simulations of 1.5 μs, each with randomized initial positions and velocities, were carried out to obtain a reliable sampling of spatial distributions.

#### 2.2.3 Peptide-Multivalent Cation Simulations

The third set of atomistic simulations was carried out to obtain the interaction potentials between amino acids and multivalent cations. We simulated the same 48 designed peptides (Table S6) as in the DNA-peptide setup, in the presence of multivalent cations, i.e., Mg(H_2_O)_6_^2+^ or CoHex^3+^. Each simulation contains one copy of all 48 peptides. The number of multivalent cations was chosen so that the charge carried by multivalent cations corresponds to three-quarters of the charge carried by all the peptides. Additionally, K^+^ and Na^+^ ions were inserted to reach a concentration of about 20 mM for both types of monovalent ions. The appropriate amount of Cl^–^ ions is used to neutralize the system. The simulations were carried out in a cubic box of 15.2 nm, each resulting in a 1.0 μs long trajectory.

#### 2.2.4 All-Atom MD Simulation Protocol

The all-atom MD simulations were conducted with the GROMACS package.^50^ The initial configurations were generated by randomly placing DNA, peptides, and/or multivalent cations in a cubic simulation box. The standard procedure of adding water molecules followed by monovalent ions is performed with tools provided by the GROMACS software. Following a short energy minimization, which removes high-energy contacts, equilibration of temperature and volume is conducted in two stages. In the first stage, the temperature is equilibrated under a Berendsen thermostat^51^ at a constant volume. In the second stage, the simulation system is subjected to a constant temperature; constant pressure equilibration which is achieved using the Berendsen thermostat and barostat.^51^ All production runs are conducted under an isothermal–isobaric ensemble at 298.15 K and 1.0 bar, realized by the velocity rescale thermostat^52^ and Parrinello–Rahman barostat.^53,54^

Depending on the composition of each subsystem, the required time of simulation differs from a few hundred to a few thousand nanoseconds. In simulations with aggregating components, i.e., DNA-Co and DNA-Peptide subsystems, the molecular configuration becomes static once equilibrium is reached as no other advanced enhanced-sampling technique is used here. Multiple trajectories started from different particle positions, and velocities are simulated for such subsystems to resolve the problem of insufficient sampling with a single trajectory. In total, we have 3 trajectories of the DNA-Co subsystem and 10 trajectories of the DNA-peptide subsystem. Other simulations were done with a single run. All trajectories are at least 1.0 μs long to allow diffusion of big DNA and peptide molecules to reach equilibrium configurations. All structural properties (RDFs, distributions of bonds, and angles) are calculated from the last 500 ns in each trajectory.

### 2.3 Derivation of the CG Effective Potential

Except for the histone core–core interactions described by the elastic network model, all other interactions between CG sites were calculated systematically and rigorously by the IMC method^37,55^. In the IMC practice, the goal is to reproduce the average structural properties of the all-atom system, such as RDFs between nonbonded CG beads and bond length and angle distributions for bonded beads. Based on the mapping rules defined earlier, these structural properties are straightforwardly calculated from the all-atom trajectories for the respective simulations. For simulations produced multiple trajectories, the structural properties are averaged over all trajectories with equal weights. The final effective potentials for the CG model are derived iteratively with the MagiC software.^55,56^ We note that for the calculated interaction terms, no other empirical parameters are introduced except those in the CHARMM27 force field. The derived potentials are tabulated and not prescribed by any specific functional form.

### 2.4 Assembling the CG NCP Model

The final CG NCP model is built by combining interaction terms from the subsystems mentioned above. Note that some of the interaction terms are available from different atomistic simulations. For example, monovalent ion-monovalent ion potentials are available from all three series of atomistic simulations; peptide–peptide interactions are available from DNA-peptide and peptide-multivalent ions simulations. Potentials between the same CG sites obtained in different simulations are typically similar but still different because of statistical uncertainty and the principal dependence of bottom-up derived effective potential on the simulation conditions (see Figure S3 and the discussion on transferability of CG potentials in Section 3.2 below). While composing the final CG NCP model, when more than one effective potential for a specific CG-site pair is available, we pick the one that corresponds best to the NCP conditions. Table S8 of the Supporting Information lists all CG-site pairs in the final CG NCP model and references to atomistic simulations from which the respective CG potential was derived.

Preliminary test runs showed that usage of only nonbonded CG DNA-histone interactions results in the overly large distance between the nucleosomal DNA and the histone core, leading to DNA detachment and unwrapping. This could happen due to two reasons. First, the CG DNA model is derived using relatively short DNAs (36 bp), which mostly keep a straight conformation in the atomistic simulations, while in an NCP, the DNA is significantly curved. Secondly, the DNA–amino acid nonbonded interaction potential might not be optimal for DNA-histone core contacts as it is derived from DNA and flexible peptides. We resolve the issue by adding harmonic bonds between the central beads of the nucleosomal DNA and their respective closest amino acid beads on the histone core. Exceptions are made for the two central beads at each end of the DNA helix to allow some “breathing” motion. The equilibrium distances of these bonds are taken from the NCP crystal structure. The same bond strength as for the bonds in histone core (5 k_B_T/Å^2^) is used. These bonds provide proper localization of nucleosomal DNA at the histone core, providing possibilities for restricted local motions. To test the effect of added DNA-histone bonds, an alternative model with fewer DNA-histone core bonds (two fewer bonds at each DNA end) is examined (Figure S4). Not surprisingly, fewer DNA-histone core bonds result in a somewhat larger NCP size due to partial DNA unwrapping. Overall, our current model is suitable for studying nucleosome–nucleosome interactions and NCP phase separation. Although DNA unwrapping is crucial in determining chromatin structure, our reference data may not at present be sufficient to model such motion. We expect that data from other simulations or experiments can be integrated into this model in the future so that both DNA unwrapping structure and dynamics can be modeled.

### 2.5 CG MD Simulations

All CGMD simulations are performed with Hamiltonian dynamics under a canonical ensemble with the LAMMPS software under the regulation of velocity rescale thermostat.^52^ Initial configurations are all generated by randomly placing components of the simulation system into cubic boxes. Simulations are initiated with a tight thermostat (temperature temping parameter being 10 ps) and a 1 fs time step. The time step is then increased to 2 fs and finally to 5 fs while relaxing the temperature damping parameter to 1000 ps. The production simulations are performed with a 5 fs time step under 310 K. The PPPM^44^ algorithm accounts for long-range electrostatic interactions.

CGMD simulations were carried out with a single NCP and in a system with 20 NCPs. A single NCP was simulated in the presence of a varying concentration of monovalent ions in order to validate the structural features of the developed CG NCP model. To study NCP–NCP interactions and their dependence on ionic conditions, a series of CG MD simulation runs were performed with 20 NCPs and monovalent (mixture of K^+^ and Na^+^, K-20NCP system), divalent (Mg(H_2_O)_6_^2+^ at 3.5, 7.1, and 10.6 mM, Mg-20NCP system), and three-valent (CoHex^3+^ at 2.3, 4.7, and 7 mM, Co-20NCP system) cations (see exact composition in Table S9). The concentrations of Mg(H_2_O)_6_^2+^ and CoHex^3+^ ions were chosen to be 0.5, 1, and 1.5 times the amount of counterions corresponding to the neutralization of the NCP negative charge. In the Mg-20NCP and Co-20NCP simulations, NCPs may aggregate due to the condensing effect of multivalent cations. Therefore, an annealing procedure is applied to improve sampling and avoid trapping in local minima. Specifically, after equilibration, the system temperature is raised from 310 K to an elevated one in 2 ns. The simulation is kept at the hot temperature for 16 ns before returning to 310 K in 2 ns. The subsequent NVT simulation is conducted for 50–100 ns depending on how fast the system reaches equilibrium. The annealing cycle is repeated 10–20 times. The elevated temperature is simulation-specific depending on the composition of each simulation box (see Table S9).

## 3 Results and Discussion

We begin with a description of the results of the all-atom MD simulations. Then, the data of the bottom-up coarse-graining will be presented, with discussions on the transferability of the interaction potentials. Finally, we describe the results of CGMD simulations of the developed CG NCP model, showing the structural properties of individual NCP and NCP aggregates in the presence of multivalent cations.

### 3.1 All-Atom Simulations

**Figure 2:**
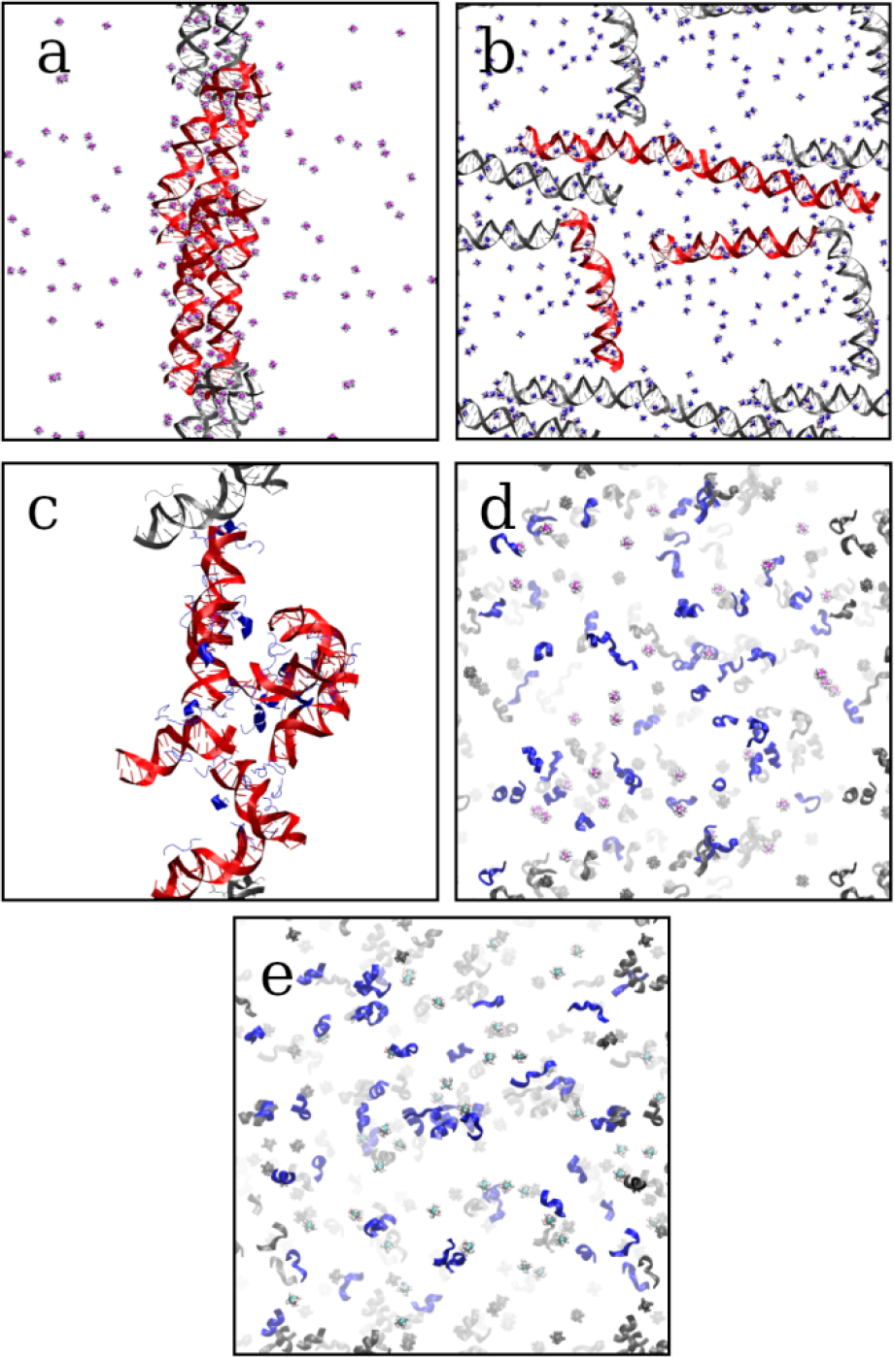
Representative snapshots of (a) DNA-Co, (b) DNA-Mg, (c) DNA-Peptide, (d) Peptide-Co, and (e) Peptide-Mg subsystems showing configurations at the end of simulations. DNA is colored red, while peptide chains are colored blue. Periodic images are colored gray.

In order to extract the effective CG potentials between the components of the CG NCP system, five subsystems were constructed and simulated under an isothermal–isobaric ensemble. The system composition of each simulation is given in Table S4. Representative snapshots of each subsystem at the end of simulations are shown in Figure 2. In agreement with the behavior predicted from experiments,^57^ DNA aggregation does not occur in the presence of Mg^2+^ ions, while the multivalent ions of charge +3 or larger induce DNA–DNA attraction demonstrated by the formation of bundles of DNA fibers.

Generation of well-converged equilibrium RDFs is critically important in structure-based coarse-graining. We quantify the RDF convergence by calculating the root mean square fluctuations (RMSF) of the nonbonded RDF in each simulation trajectory. The RMSF of the nonbonded RDF is defined as
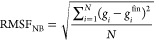
5where *g* is the nonbonded RDF calculated from a 50 ns section of the simulation, *g*^fin^ is the final nonbonded RDF calculated from the last 500 ns of the simulation, and *N* is the total number of entries of the nonbonded RDF in discrete representation.

**Figure 3:**
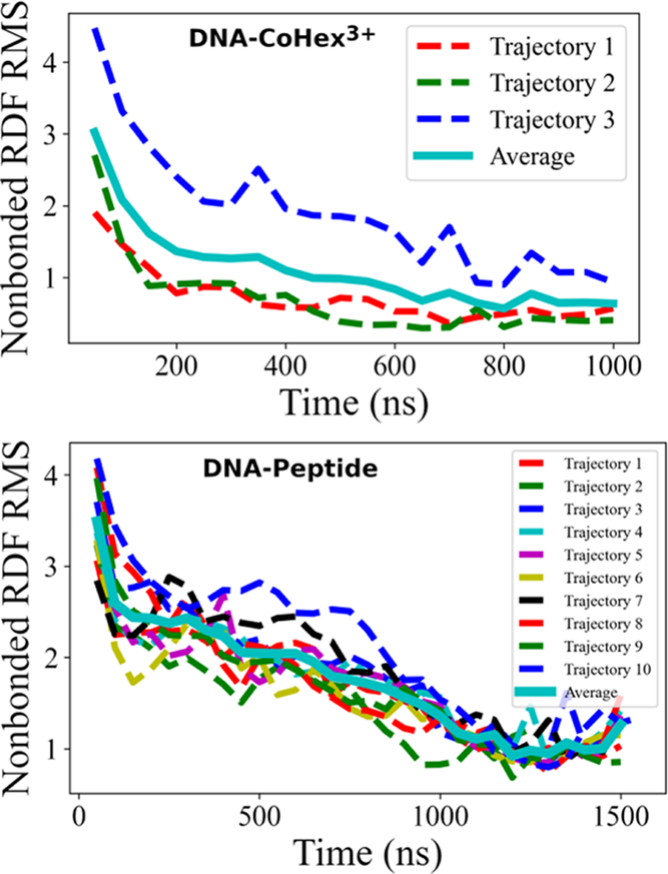
Root mean square fluctuation of block-averaged RDF relative to the final RDF for DNA-Co (top) and DNA-Peptide (bottom) subsystems. Final RDF is calculated with the last 500 ns of each trajectory.

In Figure 3, the RMSFs of nonbonded RDFs are plotted as functions of simulation time for each trajectory for the DNA-Co and DNA-Peptide subsystems (for RMSF plots of all subsystems, see Figure S5). The RDF has converged well in each trajectory, as shown by the convergence of RMSF to a low value. Notably, during the last 500 ns of each trajectory, the RMSF of RDF is stable at a low value, which validates our choice of using this section of the trajectories for the subsequent CG modeling.

### 3.2 Deriving CG Potentials

The CG effective potentials are derived by the IMC method,^37^ which takes the RDFs and bond distributions from the atomistic simulations and finds the set of potentials that reproduces the input. A detailed account of the application of the IMC method has been given earlier for the DNA-Co subsystem.^46^ Below, we give a brief description of the procedure.

For each subsystem, atomistic trajectories are first mapped to a coarse-grained description according to the mapping scheme of the specific CG model. The RDFs between nonbonded CG sites and distribution of bond lengths and angles, plotted in Figures S6–S10 for each subsystem, are computed from the last 500 ns of all trajectories. These functions (RDFs and bonded distributions) are taken as input by the Magic-3 software^55^ to conduct IMC computations. Within this approach, the system is first simulated with a trial potential. Then, comparing the computed RDFs with reference RDFs obtained in the atomistic simulations, IBI or IMC algorithms are used to compute a correction to the potential. The potential is improved iteratively until an agreement between simulated and reference RDFs is reached within the statistical error of the simulations. In the inverse procedure, the electrostatic part of the interaction is not changed and treated by the Ewald summation method,^43^ while short-range nonbonded and bonded interactions are varied.

Typically, the iterative procedure started from zero nonbonded potentials and Boltzmann inversed bonded potentials. The first 10 iterations were done according to the IBI scheme.^38^ Subsequent iterations proceed with IMC until full convergence,^37^ which typically takes 30–50 iterations. Complete sets of final short-range potentials for each subsystem are provided in Figures S6–S10, with corresponding RDFs in the Supporting Information. It should be noted that with the systematic modeling of IMC, cross correlations among interaction terms are accounted for so that the individual term does not necessarily predict the corresponding RDF. For example, the P–P nonbonded potential in the DNA-Mg system (Figure S6) is more attractive than that in the DNA-Co system (Figure S7). However, the P–P RDF in the DNA-Mg system is significantly lower as DNA molecules are not aggregating. With correlations among all interaction terms, the IMC-derived potential reproduces the reference RDF within statistical error.

**Figure 4:**
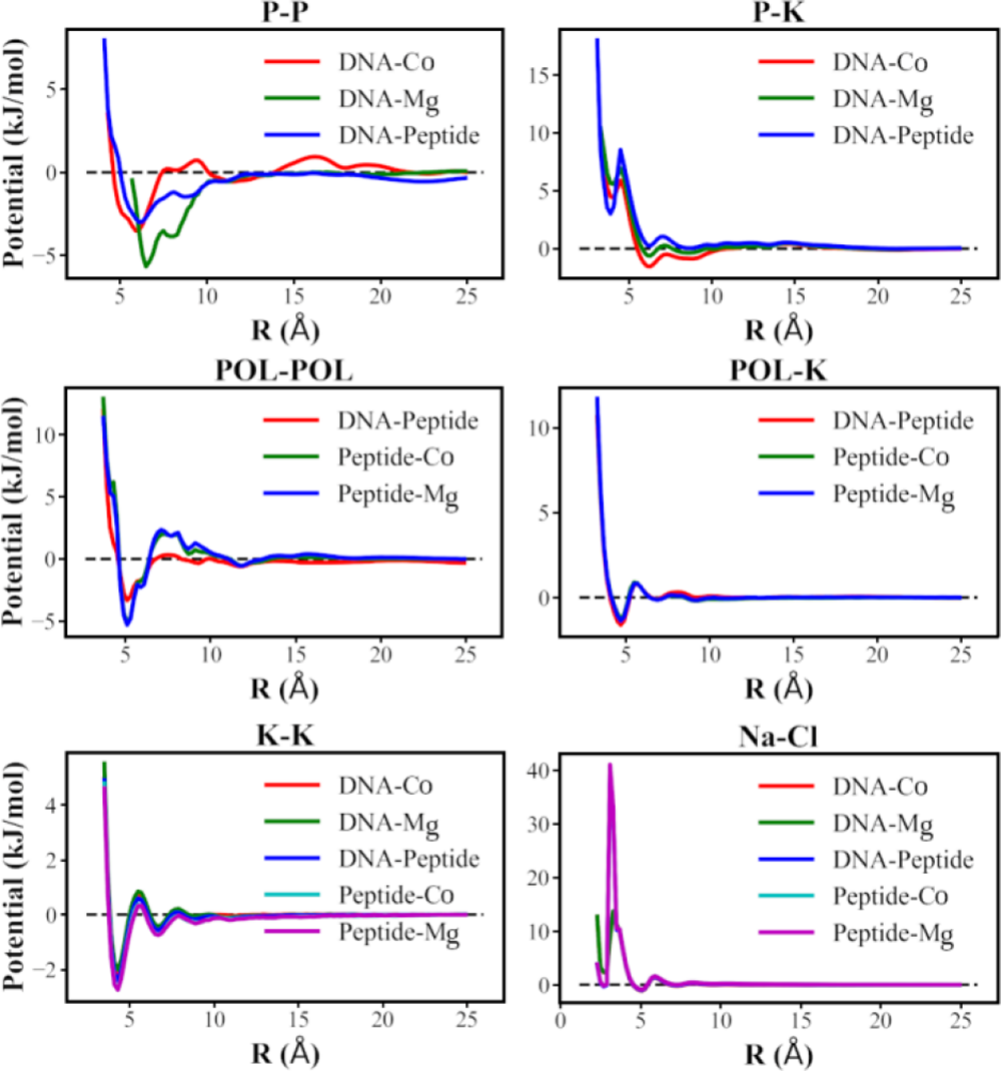
Selected short-range potential functions obtained by the IMC method from different subsystems. See Figure S3 in the Supporting Information for a complete set of such potential terms.

In our “divide and conquer” strategy, a particular interaction term can be derived from more than one subsystem. A selected set of short-range nonbonded effective potentials from different subsystems is plotted in Figure 4 for comparison. A complete comparison of all such potential terms is shown in Figure S3. Figure 4 and Figure S3 show that the difference in potential energy functions from different subsystems is minimal for terms related to ions, especially for the monovalent ions (e.g., K–K potential in Figure 4), which demonstrates the transferability of the effective potential. Most of the other potentials have the same features (peaks and wells), and the only difference is in the height of the peaks and the depth of the wells. Such terms include amino acid–amino acid (e.g., POS–POL potential in Figure 4) and amino acid–monovalent ion (e.g., POL–K potential in Figure 4).

The most significant variation of the potentials is observed for the DNA–DNA nonbonded terms, for instance, the P–P potential in Figure 4. The notable variance in DNA–DNA potentials is due to the different aggregated configurations exhibited in the simulated subsystems. CoHex^3+^ is a potent DNA condensing agent,^58,59^ while the positively charged peptides can also induce DNA aggregation.^57,60^ The question arises regarding what potentials should we use to construct the CG NCP model. As the difference in DNA–DNA potentials originates from the difference in composition of the subsystems, we follow the principle to use the effective potentials derived from the subsystem closest to our target system. Since our target system, the CG NCP, is a DNA-protein complex with DNA surrounded primarily by the histones (both core and tails), potential functions from the DNA-Peptide subsystem are preferred. The final CG NCP model is built by combining effective potentials from the subsystems according to a similar principle. The chosen combination of effective potential terms for all nonbonded interactions is summarized in Table S8.

### 3.3 Structural Features of the CG NCP Model

**Figure 5:**
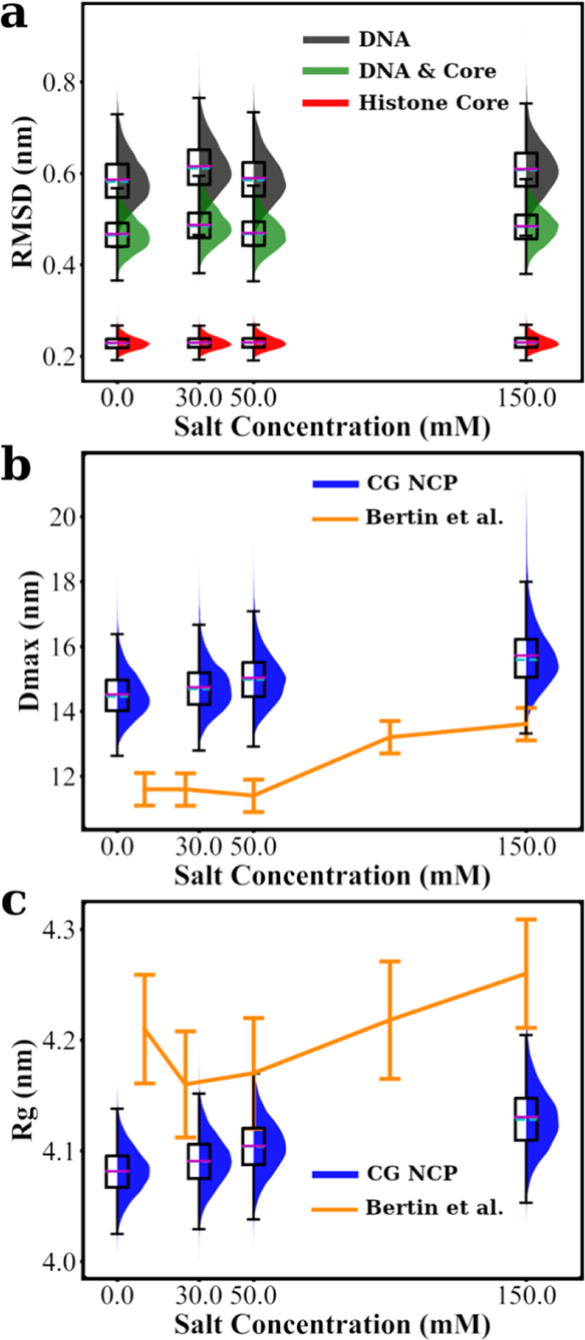
Structural features of CG NCP. Structural properties obtained in equilibrium simulation of a single NCP are presented with box plots, including the RMSD relative to the crystal structure (a), maximal dimension (*D*_max_ in b), and radius of gyration (*R*_g_ in c). The edges on each box plot represent the 25th and 75th percentile. Mean values and medians are shown as magenta and cyan lines in the boxes, respectively. Whiskers are extended for 1.5 times the interquartile range in each direction. A shaded distribution curve is plotted alongside each box to illustrate the shape of the distribution for each dataset. RMSD values are calculated and combined for DNA and histone core (as defined in Table S2). *D*_max_ and *R*_g_ are plotted together with experimental data from ref (61). All properties are shown as functions of salt concentration.

In order to validate our new CG NCP model, we first perform MD simulations of a single CG NCP at 310 K. The simulations are conducted in 30 nm cubic boxes within the canonical ensemble. In addition to the monovalent ions neutralizing the NCP charge, monovalent salt from 0 to 150 mM is added (the amounts of Na^+^ and K^+^ are equal). We examine the salt dependence of three parameters of the CG NCP structure in a single NCP system: the root mean square deviation (RMSD) relative to the reference crystal structure (PDB: 1KX5), the maximal dimension (*D*_max_), and the radius of gyration (*R*_g_) of the NCP. These properties are plotted in Figure 5 and compared with the experimental data.

First, we calculated the RMSD for DNA, histone core, and combined parts (Figure 5a). The histone core shows a low average RMSD of about 0.2 nm for all salt concentrations. It confirms the stability of the histone core structure as expected from the constructed elastic network model. The RMSD values of the nucleosomal DNA are about 0*.*6 nm. This is acceptable because the bonded interactions in DNA are derived for straight (albeit flexible) DNA, and fluctuations of the DNA structure are inherited from the underlying MD simulations. The overall RMSD for the DNA and the histone core region is about 0*.*5 nm. We conclude that the model well preserves the structure of the nucleosomal DNA and the histone core. As anticipated, there is no dependence of the RMSD on monovalent salt concentration.

To estimate the salt dependence of the overall size of the NCP within our model, taking into account the flexible tails, we calculate the values of *D*_max_ (Figure 5b) and *R*_g_ (Figure 5c). Both parameters increase slightly with the increase of salt, which can be explained by the increasing extension and mobility of the histone tails caused by weakening the tail–DNA interaction due to the screening effect of salt ions. This contribution should be the dominant contribution to salt dependence, considering the stable size of the DNA and histone core (Figure 5a). Small-angle X-ray scattering (SAXS) experiments reported *R*_g_ values of the NCP in the range 4.15–4.30 nm and *D*_max_ between 11.5 and 13.5 nm for salt concentrations in the range of 0–150 mM.^61^ The *R*_g_ values obtained in our simulations (change of mean value from 4.08 to 4.13 nm in the same salt range, 0–150 mM) agree very well with experimental data. However, in our CG NCP simulations, the mean *D*_max_ value increases from 14.5 to 15.7 nm; both numbers are larger than the experimental values, while the salt-dependent change is smaller. We can explain the difference in the *D*_max_ values by the fact that the experimental determination of the *D*_max_ relies on the quality of the SAXS spectra that are a product of several measurements with inherited experimental uncertainties and noise (subtraction of the contributions from the background solution and cell walls). In particular, higher *D*_max_ values obtained in the CG MD simulations can be explained by the contribution from the flexible histone tails sampling the space further away from NCP. In the SAXS experiments, this contribution is at the noise level due to the small contrast in electron density between the tails and the solvent, and this contribution may not be accounted for.

We can conclude from single NCP simulations that the new CG NCP model is structurally sound under the limitation of the underlying force field. Our CG NCP model agrees well with the experimental data for NCP solutions at low to physiological monovalent salt concentrations. The NCP in solution has a stable structure of the DNA and the histone core, with the histone tails collapsing on the DNA in a salt-dependent manner in agreement with the experiment.

### 3.4 NCP Aggregation in the Presence of Multivalent Cations

The primary aim of developing the new CG NCP model is to describe NCP–NCP interactions and particularly aggregation properties under different conditions. We, therefore, performed a series of CG MD simulations of 20 NCPs with different mono- or multivalent cations and background added salt (KCl and NaCl) at 15 mM (Table S9). The simulations were conducted with Hamiltonian dynamics until the equilibrium state was well sampled. When NCP aggregation occurs in simulations with multivalent ions, a simulated annealing procedure is implemented to improve sampling as described in the Methods section.

**Figure 6:**
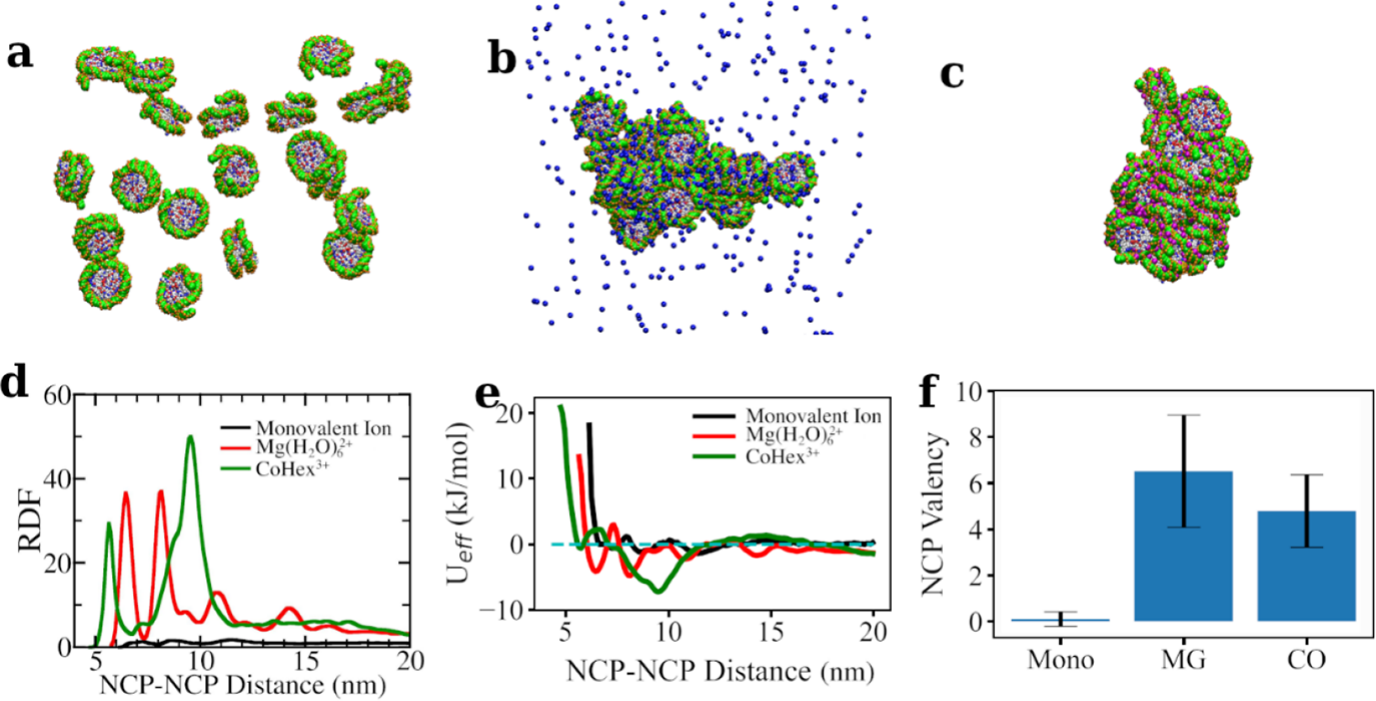
CG MD simulations of the systems with 20 NCPs and K^+^, Mg(H_2_O)_6_^2+^ (7.1 mM), and CoHex^3+^ (4.7 mM) cations. Snapshots of typical equilibrium states in K-20NCP (a), Mg-20NCP (b), and Co-20NCP (c) simulations are shown. Histone tails and monovalent ions are omitted in the snapshots for clarity. Mg(H_2_O)_6_^2+^ ions are shown as blue balls, whereas CoHex^3+^ ions are in magenta. (d) The histone core–histone core RDF. NCP–NCP interaction shows different modes as indicated by the RDF. (e) The NCP–NCP effective potentials derived from the RDFs are displayed in panel (d). (f) NCP valency is calculated according to eq 6. Error bars are standard deviations.

It is well known from in vitro experiments that multivalent ions can induce phase separation of NCP from the solution.^10,12,13^ In particular, the CoHex^3+^ ion is a highly potent condensing agent and promotes the formation of hexagonal columnar NCP phases for a wide range of ion concentrations.^10,12,13^ Even though this counterion lacks biological significance, it provides an excellent model system for validating the model’s capacity to reproduce observed behavior. Our CG model correctly reproduces the multivalent ion-induced NCP aggregation phenomenon in the MD simulations. After reaching equilibrium, NCPs stay in the solution phase when only monovalent ions are present (Figure 6a). In the presence of Mg(H_2_O)_6_^2+^ (Figure 6b) or CoHex^3+^ (Figure 6c), all 20 NCPs aggregate, forming a single cluster. This aggregation behavior is in excellent agreement with experimental data demonstrating precipitation and formation of various aggregated ordered phases of NCP.^6−13^

We analyze the structure of the NCP aggregates by plotting RDFs between the COM of the NCP histone cores (Figure 6d). In monovalent salt (K-20NCP system, black line in Figure 6d), the RDF indicates the absence of the close NCP–NCP contacts. Overall, NCPs repel each other in the presence of only monovalent ions. Mere occasional NCP contacts occur mediated by histone tail bridging and cation screening. In contrast, high-intensity peaks in the core–core RDFs are observed in the Mg-20NCP and Co-20NCP simulations. In the simulation with Mg(H_2_O)_6_^2+^ (red curve in Figure 6d), two major (at 6.45 and 8.12 nm) and two minor (at 10.8 and 14.2 nm) peaks are observed. The first peak at 6.45 nm corresponds to the stacking coordination of NCPs, where the flat surfaces of the two NCPs are in close contact. The second peak at 8.12 nm corresponds to the perpendicular coordination of the two NCPs, where DNA from one NCP is in close contact with the histone core from another NCP. The third peak at 10.8 nm represents the side-by-side positions of the NCPs with close DNA–DNA interaction, where the distance between the NCPs is approximately equal to the diameter of the particle.

The core–core RDF calculated from the Co-20NCP simulation (green curve in Figure 6d) indicates a somewhat different internal structure of the NCP aggregate. There are two major peaks at 5.65 and 9.53 nm. The first peak corresponds to the NCP stacking, 0.8 nm closer than in the Mg-20NCP simulation. This tighter NCP packing can be attributed to the higher CoHex^3+^ charge that results in the effective screening of the DNA–DNA repulsion, allowing the almost complete overlapping of the stacked NCPs. In the Mg-20NCP system, NCPs in the stack are shifted relative to each other to minimize DNA–DNA proximity. Similar to the third peak (10.8 nm) in the Mg-20NCP RDF, the second peak in the Co-20NCP system is for the side-by-side NCP contact due to the DNA–DNA interactions. In the Co-20NCP system, the perpendicular NCP coordination as well as the contact between the DNA on the NCP side and the histone core, observed in the Mg-20NCP system at 8.12 nm, is diminished. This can be explained by the ability of the CoHex^3+^ ions to cause a stronger DNA–DNA attraction than Mg(H_2_O)_6_^2+^. As a result, NCP stacking becomes overlapping, and side-by-side orientation dominates over the perpendicular NCP coordination.

Further simulations have shown that the position and magnitude of the RDF peaks depend on the multivalent ion concentrations. As shown in Figure S11, higher multivalent ion concentration generally results in the RDF peaks being shifted to the left, suggesting closer contacts between NCPs into more compact aggregates. Our future works will elaborate on the details of multivalent ion-induced NCP condensation at different ionic conditions, including an analysis of the aggregated structures.

**Figure 7:**
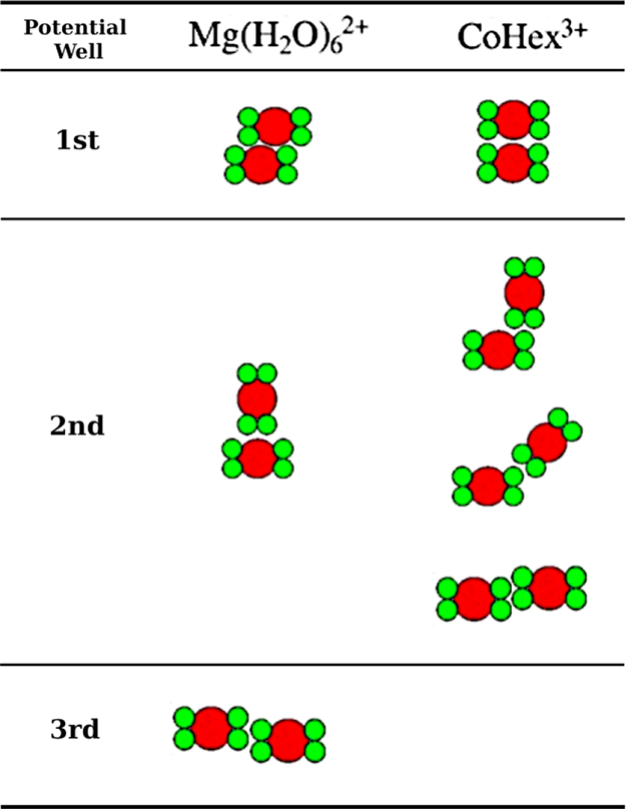
Summary of NCP–NCP binding modes in the multivalent ion-induced NCP aggregates. The most populated NCP–NCP contacts correspond to the minima in the core–core effective potential function (Figure 6e). The NCP is shown as simplified shapes representing its cross section with green circles for the DNA wrapped around the histone core (red circle).

To describe the physical picture of the NCP behavior from the core–core RDFs (Figure 6d), we used the IMC method to calculate the effective NCP–NCP pair potentials (Figure 6e). The curves in Figure 6e show dependencies of the effective NCP–NCP interaction energy as a function of the core–core distance at different ionic environments. The steep repulsive sections of the curves at a short distance show that the effective size of the NCP increases in the order Co-20NCP < Mg-20NCP < K-20NCP. When only monovalent ions are present, shallow energy minima are seen (black line in Figure 6e), revealing that NCP contacts are transient and only distant weak correlations in the NCP positions are present due to NCP–NCP repulsion. Multivalent cations screen NCP–NCP repulsion, ultimately leading to NCP–NCP attraction and aggregation. Notably, the effective potential minima determine the equilibrium NCP–NCP distances and define the internal structure of the condensed phase. In the presence of Mg(H_2_O)_6_^2+^ ions, the NCP–NCP potential shows two major minima corresponding to the two major peaks in the RDF. The first minimum at 6.45 nm, measuring the NCP–NCP face-to-face interaction, coincides with the experimentally measured value by Funke et al.^62^ The depth of this minimum (−1.0 kCal/mol) is in an acceptable range compared to the experimental value (−1.4 kCal/mol)^62^ considering the difference in Mg(H_2_O)_6_^2+^ concentration (7 mM vs 11 mM) and buffer used in the experiment. This again illustrated the advantage of the bottom-up-derived nonbonded potential in modeling NCP–NCP interactions. Interestingly, in the Co-20NCP system, the second minimum of the potential curve is deeper and broader than all other potential wells. This can be attributed to the strong DNA–DNA attraction induced by CoHex^3+^, leading to the coexistence of several types of NCP–NCP contacts with distances within the range of this potential well (illustrated in Figure 7).

**Figure 8:**
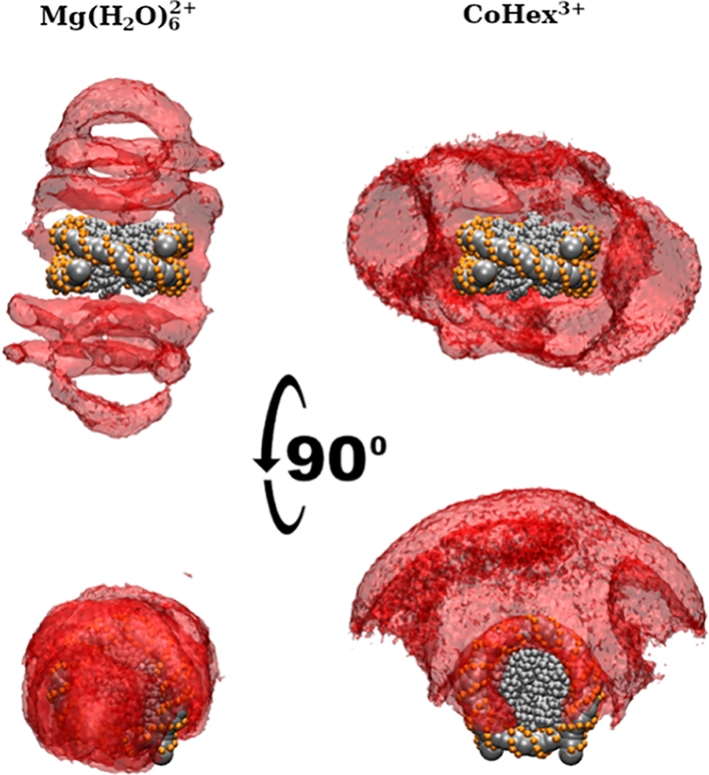
SDFs of DNA in multivalent ion-induced NCP aggregates. In the Mg-20NCP (left) and Co-20NCP (right) systems, NCP–NCP stacking is revealed by the densities above and below the central NCP. In the Mg-20NCP system, the “arcs” above and beneath the central NCP indicate a population of perpendicular NCP–NCP orientations. In the Co-20NCP system, the densities of the NCP lateral side opposite reflect contacts between DNA on the lateral surface of the wedge-shaped NCP cylinder. The absence of the SDF density at the DNA entry–exit location of the NCP indicates that most particles orient this part toward the condensate surface. See the Supporting Information for 3D animations.

The spatial distribution function (SDF) of DNA visualizes how NCPs are arranged relative to each other. Figure 8 shows that DNA densities below and above the central NCP are observed in the condensed clusters of the Mg-20NCP and Co-20NCP systems, confirming that the first peak in the RDFs (Figure 6d) corresponds to the NCP–NCP stacking. However, the face-to-side NCP–NCP contact is only seen in the Mg-20NCP system (left column of Figure 8). The perpendicular rings of DNA density above and below the reference NCP clearly show the face-to-side NCP contact. Diffuse areas on the side of the central NCP in the Co-20NCP system (right column in Figure 8) correspond to multiple orientations of the neighboring NCPs in the condensed phase, all within a distance covered by the wide second well of the core–core effective potential (green curve in Figure 6e).

In a recent paper,^35^ the concept of NCP “valency” has been suggested as a useful number counting NCP–NCP contacts in chromatin. The NCP valency is defined as the average number of NCP–NCP contacts per each NCP in the system:
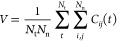
6where *i* and *j* are indices of NCP, *N*_t_ is the number of sampled snapshots, and *N*_n_ is the number of NCP in the simulation. The NCP–NCP contact (*C_ij_*(*t*)) is determined by a distance criterion
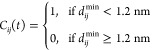
7where *d_ij_*^min^ is the minimum distance between NCP *i* and *j* (excluding histone tails). The statistics of NCP valency is shown in Figure 6f. As only occasional contacts occur in the simulation with monovalent ions, NCP valency in this system is close to zero. The high valency number reflects numerous “soft” contacts with various NCP–NCP orientations observed in the system with Mg(H_2_O)_6_^2+^. In contrast, aggregation of the NCPs caused by the CoHex^3+^ ions results in a more organized and structurally defined structure with a smaller number of distinct NCP–NCP contacts reflected by a lower valency number. The observed picture is reminiscent of the recent observation that in compact nucleosome arrays, a “trade” between entropy and energy contributions to the free energy of the condensed phase leads to the higher NCP valency in the disordered but compact arrays at physiological salt relative to the highly organized low-NCP valency folded fibers under low-salt conditions.^35^

**Figure 9:**
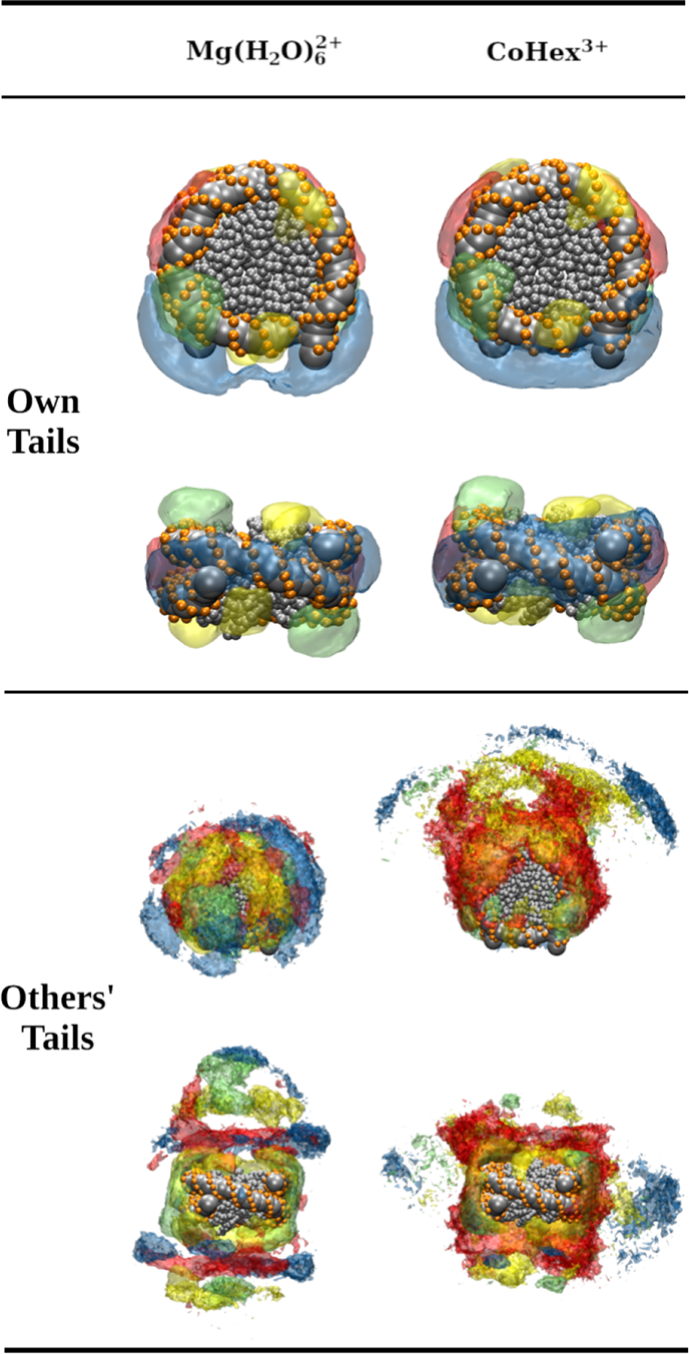
SDFs of the histone tails around the NCP calculated from the equilibrated parts of the CG MD trajectories in the Mg-20NCP and Co-20NCP systems. Threshold values used to draw the transparent isosurfaces are the same for both systems. The SDFs of the H3 tails are colored blue, the H4 tails are green, the H2A tails are yellow, and the H2B tails are red; the DNA and core histone beads of the central NCP are shown as gray spheres. The top and side views of the central NCP are shown. See the Supporting Information for the 3D animation of these distributions.

Furthermore, we examine the distribution of the histone tails around an NCP in the Mg-20NCP and Co-20NCP systems. The SDFs of the histone tails are calculated from the equilibrated parts of the CG MD trajectories. The resulting SDFs are shown in Figure 9, with isosurfaces corresponding to an equal probability of finding tail beads in the Mg-20NCP and Co-20NCP systems. Comparing the SDFs of the NCP, “Own Tails” shows a slightly larger volume of SDF in the Co-20NCP simulation. This can be explained by the tail-mediated DNA–DNA attraction in the NCP stacks; in the Co-20NCP system, the histone tails are more likely to be spread between NCPs. In addition, we can directly observe histone tail-mediated NCP–NCP interaction by plotting the SDFs of the neighboring (“Others’ Tails”) NCP tails around a central NCP. The H4 (green) and H2A (yellow) tails have close contact with neighboring NCPs both in the Mg-20NCP and Co-20NCP systems (bottom row in Figure 9). These tails mediate NCP–NCP stacking by screening DNA–DNA repulsion at the top and bottom of the NCP cylinder. The H2B tails (red) are also near the neighbors’ DNA in the condensed Co-20NCP but not in the Mg-20NCP aggregate. Last, the H3 tails are away from the neighboring NCPs in the condensed NCPs, indicating that they do not make contacts with neighbors contributing to the NCP–NCP interaction by screening the charge of their own NCP.

**Figure 10:**
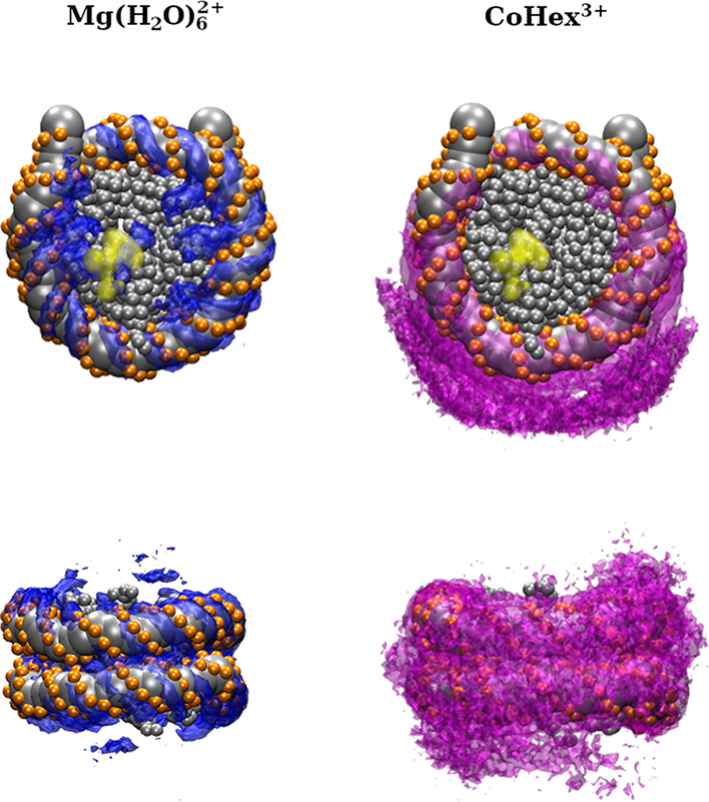
Top view (top row) and side view (bottom row) of the distribution of multivalent ions surrounding the NCP. Yellow regions in the top view panels indicate the approximate positions of the acidic patch.

Finally, the SDFs of the Mg(H_2_O)_6_^2+^ and CoHex^3+^ ions around NCP were calculated and shown as colored volumes in Figure 10. The data shows that Mg(H_2_O)_6_^2+^ ions preferably associate with the DNA and the acidic patch on the histone core, whereas CoHex^3+^ ions concentrate predominantly near the DNA. At the same time, monovalent cations are excluded from the space occupied by the multivalent ions, with CoHex^3+^ ions exerting a more substantial exchange effect than Mg(H_2_O)_6_^2+^ near the DNA (see Figure S12). This change in ionic composition near the NCP leads to an observed density of the Cl^–^ ions in the central cavity of the histone core (Figure S12), which suggests a change in the electrostatic properties of the NCP. This may serve a role in the face-to-face contact of NCPs. In the Mg-20NCP system, monovalent ions are exchanged both near the DNA and the so-called acidic patch on the surface of the HO core, resulting in uniformly lower monovalent ion concentration near the NCP. In the Co-20NCP system, there is a low probability of finding monovalent cations near the DNA, while some presence is observed near the acidic patch. Hence, we can infer that the charge screening by the Mg(H_2_O)_6_^2+^ ions is spatially spread out, while the CoHex^3+^ ions mainly neutralize nucleosomal DNA and mediate DNA–DNA attraction. This explains the differences in the NCP–NCP contacts observed in the Mg-20NCP and Co-20NCP systems.

Structural analysis of the charge–charge contacts in the NCP shows that the surface of the histone core exposed to the solvent is net negatively charged in the presence of monovalent salt, while the net charge of the core as a whole is net-positive.^14,63^ Most of the positively charged amino acids of the globular part of the histone core are neutralized by the wrapped DNA. The negative charge on the core surface is located in the two acidic patches on the two opposing flat core surfaces. Consequently, the interaction between these contact surfaces of the NCPs is repulsive in the absence of multivalent ions. From this observation, it follows that participation of the histone positively charged tails, multi- (Mg^2+^, CoHex^3+^) and monovalent (K^+^) ions are required for the DNA–DNA and the core–core interactions to become attractive and enable the close stacking of nucleosomes on top of each other illustrated in Figure 7. These interactions are indeed seen in the SDF distributions (Figures 9 and 10).

In summary, CG MD simulations show that the geometry of the NCP–NCP contacts and structure of the NCP condensed phase depend on the ionic conditions and the cation charge and nature. The lower-strength NCP–NCP attraction induced by Mg(H_2_O)_6_^2+^, with a screening effect on both nucleosomal DNA and histone core, allows multiple contact modes, including perpendicular NCP–NCP orientation, and leads to the higher NCP valency. The stronger CoHex^3+^–DNA interaction facilitates inter-nucleosome DNA–DNA attraction, limiting the number of ways of arranging the NCP around a particular NCP, producing more ordered condensed phases in agreement with experiments. This difference can explain the experimental observation^10^ that NCP precipitates induced by CoHex^3+^ are more ordered than those induced by Mg(H_2_O)_6_^2+^.

## 4 Conclusions

We have developed a CG NCP model within a bottom-up approach, establishing its parameters exclusively from atomistic simulations with no adjustable nonbonded parameters. For such a complex system as the NCP, it is extremely difficult, if not impossible, to follow a direct bottom-up approach, i.e., sampling the conformational space of several NCPs at the all-atom level with the subsequent derivation of effective CG potentials. We adopted a “divide and conquer” strategy, where the effective CG potentials are extracted from a set of all-atom MD simulations with subsets of components of the NCP subsequently integrated into the CG NCP model. MD simulations of the resulting CG NCP model have shown that the structure of the NCP is well preserved, and basic NCP structural parameters agree well with experimental SAXS data.

In developing the current CG NCP model, we intentionally did not address the influence of DNA sequence on the NCP properties. Numerous experimental studies (mainly using SAXS and X-ray diffraction methods) have proved that the phase behavior of NCP does not show sequence dependence for cell-extracted NCPs with mixed DNA sequence or for the in vitro reconstituted NCPs with sequences of high-affinity positioning “601” DNA, α-satellite DNA, and telomeric DNA (see for example refs (10, 13, 64, 65)). The present approach can be updated to include DNA sequence effects by using the IMC method to extract effective potentials defined for the DNA P and D beads for all unique two-base pair sequence combinations. However, this is outside the scope of the present work since we focus on NCP aggregation and phase behavior, which, as articulated above, do not show any sequence dependence.

Simulations with multiple NCPs have shown that the developed model correctly reproduces multivalent cation-induced NCP condensation. Further studies of NCP–NCP interaction at different ionic conditions can be directly carried out with the current CG NCP model. Additionally, a CG model of a nucleosome array with linker DNA can be built similarly using CG interaction potentials obtained in this work. Using this newly developed CG NCP model, we intend to carry out a systematic investigation of the structural properties of NCP aggregates in the presence of different concentrations of di- and trivalent ions.

## Supporting Information Available

Supporting tables and figures (PDF)
Archive including all tabulated CG potentials and Jupyter Notebook illustrating the building of CG NCP model for simulations with LAMMPS, which is also available at https://doi.org/10.5281/zenodo.5833006 (ZIP)

## References

[ref1] Zhou K.; Gaullier G.; Luger K. Nucleosome structure and dynamics are coming of age. Nat. Struct. Mol. Biol. 2019, 26, 3–13. 10.1038/s41594-018-0166-x. 30532059 PMC7386248

[ref2] Luger K.; Richmond T. J. The histone tails of the nucleosome. Curr. Opin. Genet. Dev. 1998, 8, 140–146. 10.1016/s0959-437x(98)80134-2. 9610403

[ref3] Chen P.; Li W.; Li G. Structures and functions of chromatin fibers. Annu. Rev. Biophys. 2021, 50, 95–116. 10.1146/annurev-biophys-062920-063639. 33957053

[ref4] Maeshima K.; Tamura S.; Hansen J. C.; Itoh Y. Fluid-like chromatin: Toward understanding the real chromatin organization present in the cell. Curr. Opin. Cell Biol. 2020, 64, 77–89. 10.1016/j.ceb.2020.02.016. 32283330

[ref5] Krietenstein N.; Rando O. J. Mesoscale organization of the chromatin fiber. Curr. Opin. Genet. Dev. 2020, 61, 32–36. 10.1016/j.gde.2020.02.022. 32305817

[ref6] de Frutos M.; Raspaud E.; Leforestier A.; Livolant F. Aggregation of nucleosomes by divalent cations. Biophys. J. 2001, 81, 1127–1132. 10.1016/S0006-3495(01)75769-4. 11463653 PMC1301581

[ref7] Leforestier A.; Dubochet J.; Livolant F. Bilayers of nucleosome core particles. Biophys. J. 2001, 81, 2414–2421. 10.1016/S0006-3495(01)75888-2. 11566811 PMC1301712

[ref8] Mangenot S.; Leforestier A.; Vachette P.; Durand D.; Livolant F. Salt-induced conformation and interaction changes of nucleosome core particles. Biophys. J. 2002, 82, 345–356. 10.1016/S0006-3495(02)75399-. 11751321 PMC1302474

[ref9] Mangenot S.; Leforestier A.; Durand D.; Livolant F. X-ray diffraction characterization of the dense phases formed by nucleosome core particles. Biophys. J. 2003, 84, 2570–2584. 10.1016/S0006-3495(03)75062-0. 12668465 PMC1302823

[ref10] Berezhnoy N. V.; Liu Y.; Allahverdi A.; Yang R.; Su C.-J.; Liu C. F.; Korolev N.; Nordenskiöld L. The influence of ionic environment and histone tails on columnar order of nucleosome core particles. Biophys. J. 2016, 110, 1720–1731. 10.1016/j.bpj.2016.03.016. 27119633 PMC4850351

[ref11] Mangenot S.; Leforestier A.; Durand D.; Livolant F. Phase diagram of nucleosome core particles. J. Mol. Biol. 2003, 333, 907–916. 10.1016/j.jmb.2003.09.015. 14583189

[ref12] Bertin A.; Mangenot S.; Renouard M.; Durand D.; Livolant F. Structure and phase diagram of nucleosome core particles aggregated by multivalent cations. Biophys. J. 2007, 93, 3652–3663. 10.1529/biophysj.107.108365. 17693471 PMC2072050

[ref13] Livolant F.; Mangenot S.; Leforestier A.; Bertin A.; de Frutos M.; Raspaud E.; Durand D. Are liquid crystalline properties of nucleosomes involved in chromosome structure and dynamics?. Philos. Trans. R. Soc., A 2006, 364, 2615–2633. 10.1098/rsta.2006.1843. 16973479

[ref14] Korolev N.; Lyubartsev A. P.; Nordenskiöld L. A systematic analysis of nucleosome core particle and nucleosome-nucleosome stacking structure. Sci. Rep. 2018, 8, 1543 10.1038/s41598-018-19875-0. 29367745 PMC5784010

[ref15] Woodcock C. L.; Grigoryev S. A.; Horowitz R. A.; Whitaker N. A chromatin folding model that incorporates linker variability generates fibers resembling the native structures. Proc. Natl. Acad. Sci. U. S. A. 1993, 90, 9021–9025. 10.1073/pnas.90.19.9021. 8415647 PMC47493

[ref16] Ehrlich L.; Münkel C.; Chirico G.; Langowski J. A Brownian dynamics model for the chromatin fiber. Bioinformatics 1997, 13, 271–279. 10.1093/bioinformatics/13.3.271. 9183532

[ref17] Katritch V.; Bustamante C.; Olson W. K. Pulling chromatin fibers: Computer simulations of direct physical micromanipulations. J. Mol. Biol. 2000, 295, 29–40. 10.1006/jmbi.1999.3021. 10623506

[ref18] Ben-Haïm E.; Lesne A.; Victor J. M. Chromatin: a tunable spring at work inside chromosomes. Phys. Rev. E 2001, 64, 051921 10.1103/PhysRevE.64.051921. 11735982

[ref19] Schiessel H.; Gelbart W. M.; Bruinsma R. DNA folding: structural and mechanical properties of the two-angle model for chromatin. Biophys. J. 2001, 80, 1940–1956. 10.1016/s0006-3495(01)76164-4. 11259307 PMC1301383

[ref20] Wedemann G.; Langowski J. Computer simulation of the 30-nanometer chromatin fiber. Biophys. J. 2002, 82, 2847–2859. 10.1016/S0006-3495(02)75627-0. 12023209 PMC1302074

[ref21] Mergell B.; Everaers R.; Schiessel H. Nucleosome interactions in chromatin: fiber stiffening and hairpin formation. Phys. Rev. E 2004, 70, 011915 10.1103/PhysRevE.70.011915. 15324096

[ref22] Kepper N.; Foethke D.; Stehr R.; Wedemann G.; Rippe K. Nucleosome geometry and internucleosomal interactions control the chromatin fiber conformation. Biophys. J. 2008, 95, 3692–3705. 10.1529/biophysj.107.121079. 18212006 PMC2553103

[ref23] Stehr R.; Kepper N.; Rippe K.; Wedemann G. The effect of internucleosomal interaction on folding of the chromatin fiber. Biophys. J. 2008, 95, 3677–3691. 10.1529/biophysj.107.120543. 18658212 PMC2553136

[ref24] Luger K.; Mader A. W.; Richmond R. K.; Sargent D. F.; Richmond T. J. Crystal structure of the nucleosome core particle at 2.8 Å resolution. Nature 1997, 389, 251–260. 10.1038/38444. 9305837

[ref25] Davey C. A.; Sargent D. F.; Luger K.; Maeder A. W.; Richmond T. J. Solvent mediated interactions in the structure of nucleosome core particle at 1.9 Å resolution. J. Mol. Biol. 2002, 319, 1097–1113. 10.1016/S0022-2836(02)00386-8. 12079350

[ref26] Korolev N.; Lyubartsev A. P.; Nordenskiöld L. Computer modeling demonstrates that electrostatic attraction of nucleosomal DNA is mediated by histone tails. Biophys. J. 2006, 90, 4305–4316. 10.1529/biophysj.105.080226. 16565063 PMC1471847

[ref27] Beard D. A.; Schlick T. Computational modeling predicts the structure and dynamics of chromatin fiber. Structure 2001, 9, 105–114. 10.1016/s0969-2126(01)00572-x. 11250195

[ref28] Beard D. A.; Schlick T. Modeling salt-mediated electrostatics of macromolecules: the discrete surface charge optimization algorithm and its application to the nucleosome. Biopolymers 2001, 58, 106–115. 10.1002/1097-0282(200101)58:1<106::AID-BIP100>3.0.CO;2-#. 11072233

[ref29] Zhang Q.; Beard D. A.; Schlick T. Constructing irregular surfaces to enclose macromolecular complexes for mesoscale modeling using the discrete surface charge optimization (DiSCO) algorithm. J. Comput. Chem. 2003, 24, 2063–2074. 10.1002/jcc.10337. 14531059

[ref30] Sun J.; Zhang Q.; Schlick T. Electrostatic mechanism of nucleosomal array folding revealed by computer simulation. Proc. Natl. Acad. Sci. U. S. A. 2005, 102, 8180–8185. 10.1073/pnas.0408867102. 15919827 PMC1140479

[ref31] Fan Y.; Korolev N.; Lyubartsev A. P.; Nordenskiöld L. An advanced coarse-grained nucleosome core particle model for computer simulations of nucleosome-nucleosome interactions under varying ionic conditions. PLoS One 2013, 8, e54228 10.1371/journal.pone.0054228. 23418426 PMC3572162

[ref32] Voltz K.; Trylska J.; Tozzini V.; Kurkal-Siebert V.; Langowski J.; Smith J. Coarse-grained force field for the nucleosome from self-consistent multiscaling. J. Comput. Chem. 2008, 29, 1429–1439. 10.1002/jcc.20902. 18270964

[ref33] Moller J.; Lequieu J.; de Pablo J. J. The free energy landscape of internucleosome interactions and its relation to chromatin fiber structure. ACS Cent. Sci. 2019, 5, 341–348. 10.1021/acscentsci.8b00836. 30834322 PMC6396382

[ref34] Lequieu J.; Schwartz D. C.; de Pablo J. J. In silico evidence for sequence-dependent nucleosome sliding. Proc. Natl. Acad. Sci. U. S. A. 2017, 114, E9197–E9205. 10.1073/pnas.1705685114. 29078285 PMC5676884

[ref35] Farr S. E.; Woods E. J.; Joseph J. A.; Garaizar A.; Collepardo-Guevara R. Nucleosome plasticity is a critical element of chromatin liquid-liquid phase separation and multivalent nucleosome interactions. Nat. Commun. 2021, 12, 2883 10.1038/s41467-021-23090-3. 34001913 PMC8129070

[ref36] Voltz K.; Trylska J.; Calimet N.; Smith J. C.; Langowski J. Unwrapping of nucleosomal DNA ends: A multiscale molecular dynamics study. Biophys. J. 2012, 102, 849–858. 10.1016/j.bpj.2011.11.4028. 22385856 PMC3283802

[ref37] Lyubartsev A. P.; Mirzoev A.; Chen L.-J.; Laaksonen A. Systematic coarse-graining of molecular models by the Newton inversion method. Faraday Discuss. 2010, 144, 43–56. 10.1039/B901511F. 20158022

[ref38] Reith D.; Putz M.; Muller-Plathe F. Derived effective mesoscale potentials from atomistic simulations. J. Comput. Chem. 2003, 24, 1624–1636. 10.1002/jcc.10307. 12926006

[ref39] Hart K.; Foloppe N.; Baker C. M.; Denning E. J.; Nilsson L.; MacKerell A. D. Jr. Optimization of the CHARMM additive force field for DNA: Improved treatment of the BI/BII conformational equilibrium. J. Chem. Theory Comput. 2012, 8, 348–362. 10.1021/ct200723y. 22368531 PMC3285246

[ref40] Sun T.; Mirzoev A.; Korolev N.; Lyubartsev A. P.; Nordenskiöld L. All-atom MD simulation of DNA condensation using ab initio derived force field parameters of cobalt(III)-hexammine. J. Phys. Chem. B 2017, 121, 7761–7770. 10.1021/acs.jpcb.7b03793. 28746805

[ref41] Minhas V.; Sun T.; Mirzoev A.; Korolev N.; Lyubartsev A. P.; Nordenskiöld L. Modeling DNA flexibility: Comparison of force fields from atomistic to multiscale levels. J. Phys. Chem. B 2020, 124, 38–49. 10.1021/acs.jpcb.9b09106. 31805230

[ref42] Krainer G.; Welsh T. J.; Joseph J. A.; Espinosa J. R.; Wittmann S.; de Csilléry E.; Sridhar A.; Toprakcioglu Z.; Gudiškytė G.; Czekalska M. A.; Arter W. E.; Guillén-Boixet J.; Franzmann T. M.; Qamar S.; George-Hyslop P. S.; Hyman A. A.; Collepardo-Guevara R.; Alberti S.; Knowles T. P. J. Reentrant liquid condensate phase of proteins is stabilized by hydrophobic and non-ionic interactions. Nat. Commun. 2021, 12, 1085 10.1038/s41467-021-21181-9. 33597515 PMC7889641

[ref43] Ewald P. P. Die Berechnung optischer und elektrostatischer Gitterpotentiale. Ann. Phys. 1921, 369, 253–287. 10.1002/andp.19213690304.

[ref44] Hockney R. W.; Eastwood J. W., Computer simulation using particles; Taylor & Francis, Inc.: Bristol, PA, USA, 1988; p 564.

[ref45] Depken M.; Schiessel H. Nucleosome shape dictates chromatin fiber structure. Biophys. J. 2009, 96, 777–784. 10.1016/j.bpj.2008.09.055. 19186120 PMC2716636

[ref46] Sun T.; Mirzoev A.; Minhas V.; Korolev N.; Lyubartsev A. P.; Nordenskiöld L. A multiscale analysis of DNA phase separation: from atomistic to mesoscale level. Nucleic Acids Res. 2019, 47, 5550–5562. 10.1093/nar/gkz377. 31106383 PMC6582353

[ref47] Korolev N.; Allahverdi A.; Yang Y.; Fan Y.; Lyubartsev A. P.; Nordenskiold L. Electrostatic origin of salt-induced nucleosome array compaction. Biophys. J. 2010, 99, 1896–1905. 10.1016/j.bpj.2010.07.017. 20858435 PMC2941033

[ref48] Yoo J.; Aksimentiev A. Improved parametrization of Li^+^, Na^+^, K^+^, and Mg^2+^ ions for all-atom Molecular Dynamics simulations of nucleic acid systems. J. Phys. Chem. Lett. 2012, 3, 45–50. 10.1021/jz201501a.

[ref49] Grotz K. K.; Schwierz N. Optimized magnesium force field parameters for biomolecular simulations with accurate solvation, ion-binding, and water-exchange properties in SPC/E, TIP3P-fb, TIP4P/2005, TIP4P-Ew, and TIP4P-D. J. Chem. Theory Comput. 2022, 18, 526–537. 10.1021/acs.jctc.1c00791. 34881568 PMC8757469

[ref50] Abraham M. J.; Murtola T.; Schulz R.; Páll S.; Smith J. C.; Hess B.; Lindahl E. GROMACS: High performance molecular simulations through multi-level parallelism from laptops to supercomputers. SoftwareX 2015, 1-2, 19–25. 10.1016/j.softx.2015.06.001.

[ref51] Berendsen H. J. C.; Postma J. P. M.; van Gunsteren W. F.; DiNola A.; Haak J. R. Molecular dynamics with coupling to an external bath. J. Chem. Phys. 1984, 81, 3684–3690. 10.1063/1.448118.

[ref52] Bussi G.; Donadio D.; Parrinello M. Canonical sampling through velocity rescaling. J. Chem. Phys. 2007, 126, 014101 10.1063/1.2408420. 17212484

[ref53] Parrinello M.; Rahman A. Crystal structure and pair potentials: A Molecular-Dynamics study. Phys. Rev. Lett. 1980, 45, 1196–1199. 10.1103/PhysRevLett.45.1196.

[ref54] Parrinello M.; Rahman A. Polymorphic transitions in single crystals: A new molecular dynamics method. J. Appl. Phys. 1981, 52, 7182–7190. 10.1063/1.328693.

[ref55] Mirzoev A.; Nordenskiöld L.; Lyubartsev A. Magic v.3: An integrated software package for systematic structure-based coarse-graining. Comput. Phys. Commun. 2019, 237, 263–273. 10.1016/j.cpc.2018.11.018.

[ref56] Mirzoev A.; Lyubartsev A. P. MagiC: Software package for multiscale modeling. J. Chem. Theory Comput. 2013, 9, 1512–1520. 10.1021/ct301019v. 26587613

[ref57] Bloomfield V. A. DNA condensation by multivalent cations. Biopolymers 1997, 44, 269–282. 10.1002/(SICI)1097-0282(1997)44:3<269::AID-BIP6>3.0.CO;2-T. 9591479

[ref58] Korolev N.; Berezhnoy N. V.; Eom K. D.; Tam J. P.; Nordenskiöld L. A universal description for the experimental behavior of salt-(in)dependent oligocation-induced DNA condensation. Nucleic Acids Res. 2012, 40, 2808–2821. 10.1093/nar/gks214. 22563605 PMC3729243

[ref59] Matulis D.; Rouzina I.; Bloomfield V. A. Thermodynamics of DNA binding and condensation: isothermal titration calorimetry and electrostatic mechanism. J. Mol. Biol. 2000, 296, 1053–1063. 10.1006/jmbi.1999.3470. 10686103

[ref60] Korolev N.; Vorontsova O. V.; Nordenskiöld L. Physicochemical analysis of electrostatic foundation for DNA-protein interactions in chromatin transformations. Prog. Biophys. Mol. Biol. 2007, 95, 23–49. 10.1016/j.pbiomolbio.2006.11.003. 17291569

[ref61] Bertin A.; Renouard M.; Pedersen J. S.; Livolant F.; Durand D. H3 and H4 histone tails play a central role in the interactions of recombinant NCPs. Biophys. J. 2007, 92, 2633–2645. 10.1529/biophysj.106.093815. 17237203 PMC1864844

[ref62] Funke J. J.; Ketterer P.; Lieleg C.; Schunter S.; Korber P.; Dietz H. Uncovering the forces between nucleosomes using DNA origami. Sci. Adv. 2016, 2, e1600974 10.1126/sciadv.1600974. 28138524 PMC5262459

[ref63] Cherstvy A. G. Electrostatic interactions in biological DNA-related systems. Phys. Chem. Chem. Phys. 2011, 13, 9942–9968. 10.1039/c0cp02796k. 21431196

[ref64] Shi X.; Prasanna C.; Soman A.; Pervushin K.; Nordenskiöld L. Dynamic networks observed in the nucleosome core particles couple the histone globular domains with DNA. Commun. Biol. 2020, 3, 639 10.1038/s42003-020-01369-3. 33128005 PMC7599221

[ref65] Eltsov M.; Grewe D.; Lemercier N.; Frangakis A.; Livolant F.; Leforestier A. Nucleosome conformational variability in solution and in interphase nuclei evidenced by cryo-electron microscopy of vitreous sections. Nucleic Acids Res. 2018, 46, 9189–9200. 10.1093/nar/gky670. 30053160 PMC6158616

