# Supplementary material for: A Bottom-Up Coarse-Grained Model for Nucleosome–Nucleosome Interactions with Explicit Ions
Source: J Chem Theory Comput. 2022 May 17;18(6):3948–60. doi: 10.1021/acs.jctc.2c00083 (PMC9202350; doi:10.1021/acs.jctc.2c00083)
Supplement: Supplementary file 1 — ct2c00083_si_001.pdf [file ct2c00083_si_001.pdf]

Supporting Information for

A Bottom-Up Coarse-Grained Model for Nucleosome-Nucleosome Interactions with Explicit Ions

Tiedong Sun,† Vishal Minhas,† Alexander Mirzoev,† Nikolay Korolev,† Alexander P. Lyubartsev, *‡ and Lars Nordenskiöld*,†

†*School of Biological Sciences, Nanyang Technological University, Singapore*

‡*Department of Materials and Environmental Chemistry, Stockholm University, Stockholm*

*E-mail: alexander.lyubartsev@mmk.su.se; larsnor@ntu.edu.sg

Supporting Tables

Table S1: **Grouping of the amino acids for CG mapping**. Amino acid residues are divided into five CG bead types in the NCP model. Mapping amino acids into CG types and the integer charge of each CG type are listed.

Amino acid	CG type	Charge (e)
ARG<br>LYS	Positive (POS)	+1.0
GLU<br>ASP	Negative (NEG)	−1.0
ASN<br>CYS<br>GLN<br>HIS<br>THR<br>SER	Polar (POL)	0.0
ALA<br>ILE<br>LEU<br>MET<br>PHE<br>PRO<br>TYR<br>TRP<br>VAL	Non-polar (NPL)	0.0
GLY	Glycine (GLY)	0.0

Table S2: Assignment of the amino acids to histone tail and core regions in the CG NCP model.

Histone	Tail region(s)	Core region
H3	ALA1-PRO43	GLY44-ALA135
H4	SER1-ASP24	ASN25-GLY102
H2A	SER1-GLN24, PRO117-LYS128	PHE25-LEU116
H2B	PRO1-GLU35	SER36-LYS125

Table S3: **Effective radii of histone core bead types to account for the excluded volume interaction**.

Core bead type	Effective radius σ (Å)
GLY	4.0
NEG	4.0
POS	5.2
NPL	4.0
POL	4.0

Table S4: Setup of the all-atom MD simulations. Numbers in the columns to the ions indicate the number of ions in respective simulations.

System	DNA	Peptide	$CoHex^{3+}$	$Mg(H_2O)_6^{2+}$	K^+	Na^+	Cl^-	Box size (nm^3)	Trajectories
DNA + $CoHex^{3+}$	4×36 bp	-	140	-	140	95	375	16.5^3	3×1.0 μs
DNA + $Mg(H_2O)_6^{2+}$	4×36 bp	-	-	140	210	165	375	16.6^3	1×1.0 μs
DNA + Peptides	8×16 bp	48×8 AA	-	-	134	261	275	15.1^3	10×1.5 μs
Peptide + $CoHex^{3+}$	-	48×8 AA	30	-	47	48	305	15.2^3	1×1.5 μs
Peptide + $Mg(H_2O)_6^{2+}$	-	48×8 AA	-	45	47	48	305	15.1^3	1×2.0 μs

Table S5: **DNA sequences in the all-atom MD simulations of the DNA-multivalent ion systems**.

No	Sequence
1	5’-ATTAATGGAACGTAGCATATTCTTCAAGTTGTCACG-3’
2	5’-CAAAACCTGATGCACACTGTAACATGAGATCCCGCG-3’
3	5’-TCGGCTTATAGAGGGCCAGCTCGTATCGACGGACCG-3’
4	5’-GCTAGTACCCCACCAATTTAGGCGAAAGGAGTCTGC-3’

Table S6: Amino acid sequences used in the DNA + peptide all-atom MD simulations.

No.	Peptide sequence	No.	Peptide sequence
1	AKRHRKVL	25	PKKGSKKA
2	AKSAPAPK	26	PKKTESSK
3	APKKGSKK	27	PRKQLATK
4	ARTKQTAR	28	PRQQLATH
5	ATGGVKKP	29	QLATKPRK
6	ESSKPKKT	30	RHEKVLRD
7	GAKRHRDV	31	RHRKGHAK
8	GAKRHRKV	32	RHRKVLRD
9	GGAKRHRK	33	RKSAPKAA
10	GGKAPRKQ	34	RKSTGGKA
11	GGKTRAKA	35	SAPAPKKG
12	GKGLGKGG	36	SAPATGRV
13	HRKVLRDQ	37	SGRGKGGK
14	KAARKSAP	38	SGRGKQGG
15	KAKTRSSR	39	SKDAPKHG
16	KAVTKTQK	40	SKKAPKKG
17	KGGEGLHK	41	SRHGKQGG
18	KGGKGLGK	42	SSKSAKSK
19	KKAVTKTQ	43	TESSKSAK
20	KKDGKERR	44	TEVVKSAL
21	KKDGKKRR	45	TKTQKKDG
22	KSAPATGR	46	VKKPATGG
23	KTQEKDGK	47	VLRDRHEK
24	KTQKKDGK	48	VLRDSGRG

Table S7: Sequences of the double-helical DNA used in the DNA + peptide all-atom MD simulations.

No.	Sequence
1	5’-TAATGGAACGTAGCAT-3’
2	5’-ATTCTTCAAGTTGTCA-3’
3	5’-AAACCTGATGCACACT-3’
4	5’-GTAACATGAGATCCCG-3’
5	5’-GGCTTATAGAGGGCCA-3’
6	5’-GCTCGTATCGACGGAC-3’
7	5’-TAGTACCCCACCAATT-3’
8	5’-TAGGCGAAAGGAGTCT-3’

Table S8: Construction of the CG NCP model. The interaction potentials of the CG NCP model were determined by the IMC method using the data from several all-atom MD simulations of the simplified subsystems (see Table S4). Here blue cells indicate the presence of the given interaction terms in the specific subsystem. Ticks indicate the interaction potentials used in the CG NCP model. An exception is made for the potential in the histone core since it is structurally well-defined and modeled by an elastic network and excluded volume interactions (see main text for details).

Potential term		DNA-Co	DNA-Mg	DNA-Peptide	Peptide-Co	Peptide-Mg
Non-bonded	D-D			✓		
	D-P			✓		
	D-POL			✓		
	D-NPL			✓		
	D-POS			✓		
	D-NEG			✓		
	D-GLY			✓		
	D-Co	✓				
	D-Mg		✓			
	D-K			✓		
	D-Na			✓		
	D-Cl			✓		
	P-P			✓		
	P-POL			✓		
	P-NPL			✓		
	P-POS			✓		
	P-NEG			✓		
	P-GLY			✓		
	P-Co	✓				
	P-Mg		✓			
	P-K			✓		
	P-Na			✓		
	P-Cl			✓		
	POL-POL			✓		
	POL-NPL			✓		
	POL-POS			✓		
	POL-NEG			✓		
	POL-GLY			✓		
	POL-Co				✓	
	POL-Mg					✓
	POL-K			✓		
	POL-Na			✓		
	POL-Cl			✓		
	NPL-NPL			✓		
	NPL-POS			✓		
	NPL-NEG			✓		
	NPL-GLY			✓		
	NPL-Co				✓	
	NPL-NG					✓

	NPL-K			✓		
	NPL-Na			✓		
	NPL-Cl			✓		
	POS-POS			✓		
	POS-NEG			✓		
	POS-GLY			✓		
	POS-Co				✓	
	POS-Mg					✓
	POS-K			✓		
	POS-Na			✓		
	POS-Cl			✓		
	NEG-NEG			✓		
	NEG-GLY			✓		
	NEG-Co				✓	
	NEG-Mg					✓
	NEG-K			✓		
	NEG-Na			✓		
	NEG-Cl			✓		
	GLY-GLY			✓		
	GLY-Co				✓	
	GLY-Mg					✓
	GLY-K			✓		
	GLY-Na			✓		
	GLY-Cl			✓		
	Co-Co	✓				
	Co-K	✓				
	Co-Na	✓				
	Co-Cl	✓				
	Mg-Mg		✓			
	Mg-K		✓			
	Mg-Na		✓			
	Mg-Cl		✓			
	K-K	✓				
	K-Na	✓				
	K-Cl	✓				
	Na-Na	✓				
	Na-Cl	✓				
	Cl-Cl	✓				
	Core-Core	Excluded Volume (Eqn 4)				
Bonds	D-P	✓				
	P-P (strand)	✓				
	D-D	✓				
	P-P (groove)	✓				
	Peptide Chain			✓		
	DNA-Core	Elastic Network (see main text)				
	Core-Core	Elastic Network (see main text)				

Angles	P-D-P	✓				
	P-P-P	✓				
	D-D-D	✓				
	X-PRO-X			✓		
	X-GLY-X			✓		
	Other X-X-X			✓		

Table S9: Setups of the multi-NCP CG simulations. Numbers in the columns to the ions indicate the number of ions in respective simulations.

Simulation	Box size (nm)	NCP	Mg^{2+}	$CoHex^{3+}$	K^+	Na^+	Cl^-	High temp (K)*
**NCP + Monovalent ions**	70.0	20	0	0	4565	4565	6210	N.A.
**NCP + $Mg(H_2O)_6^{2+}$**	70.0	20	730	0	3105	3105	4750	520
	70.0	20	1460	0	3105	3105	6210	550
	70.0	20	2190	0	3105	3105	7670	550
**NCP + $CoHex^{3+}$**	70.0	20	0	485	3105	3105	4745	360
	70.0	20	0	970	3105	3105	6200	600
	70.0	20	0	1460	3105	3105	7670	700

*Temperatures used in the annealing procedure.

Supporting Figures

Figure S1

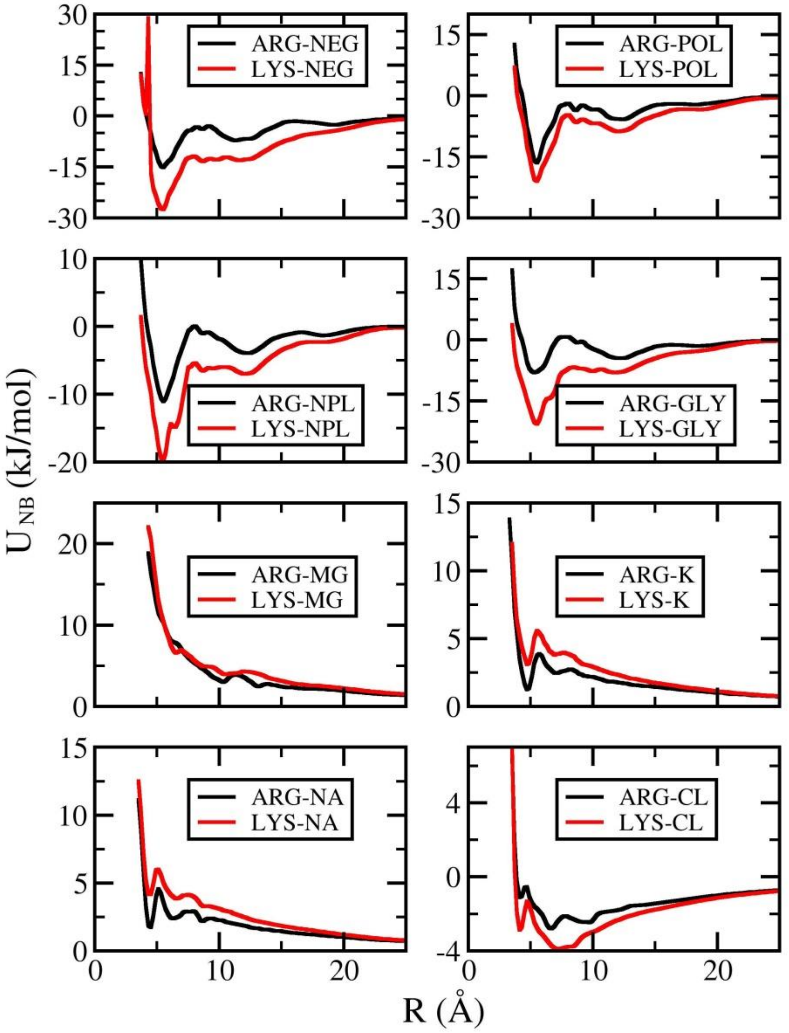

Figure S1: Non-bonded interaction potential if arginine and lysine were treated as respective bead types. In one of the subsystems, Peptide-Mg, arginine, and lysine are assigned to two nonidentical CG bead types before calculating the effective potential with IMC. The non-bonded effective potential (sum of short-range and electrostatic potentials) is compared between arginine (black curves) and lysine (red curves) beads. In general, arginine and lysine display similar interactions with other CG beads, validating the bead type definition in the CG NCP model.

Figure S2

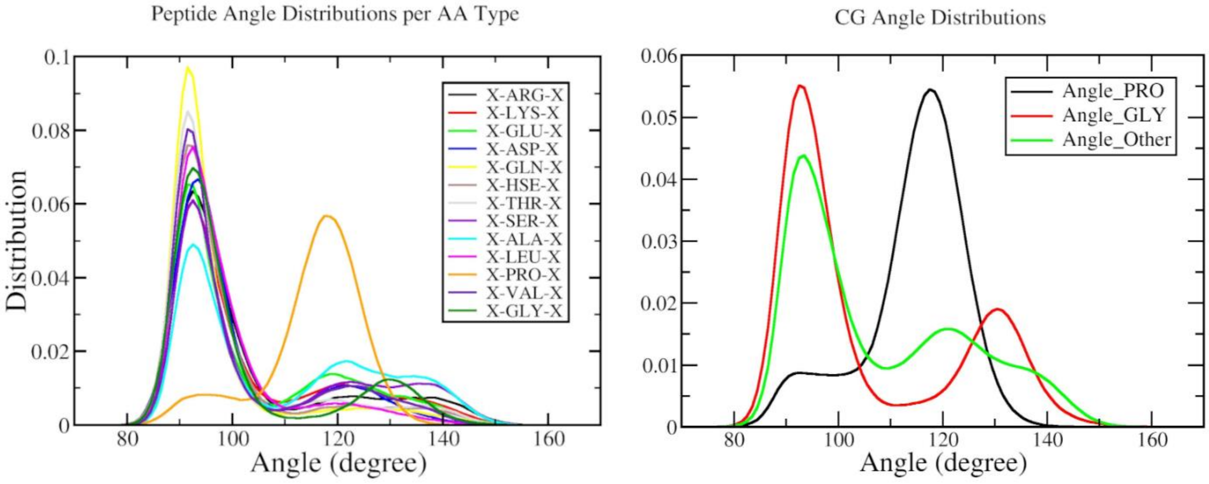

Figure S2: **Normalized distribution of CG angle types.** The angle distribution of every X-AA-X, where AA is any amino acid residue, is plotted on the left. CG angle distributions are shown on the right. Proline presents distinctive X-AA-X angle distributions than other amino acids. Glycine shows a peak between 120 and 140 degrees instead of a plateau shown by other amino acids. Hence three angle types are used in the CG NCP model along the histone tail chains, namely Angle PRO, Angle GLY, and Angle Other.

Figure S3

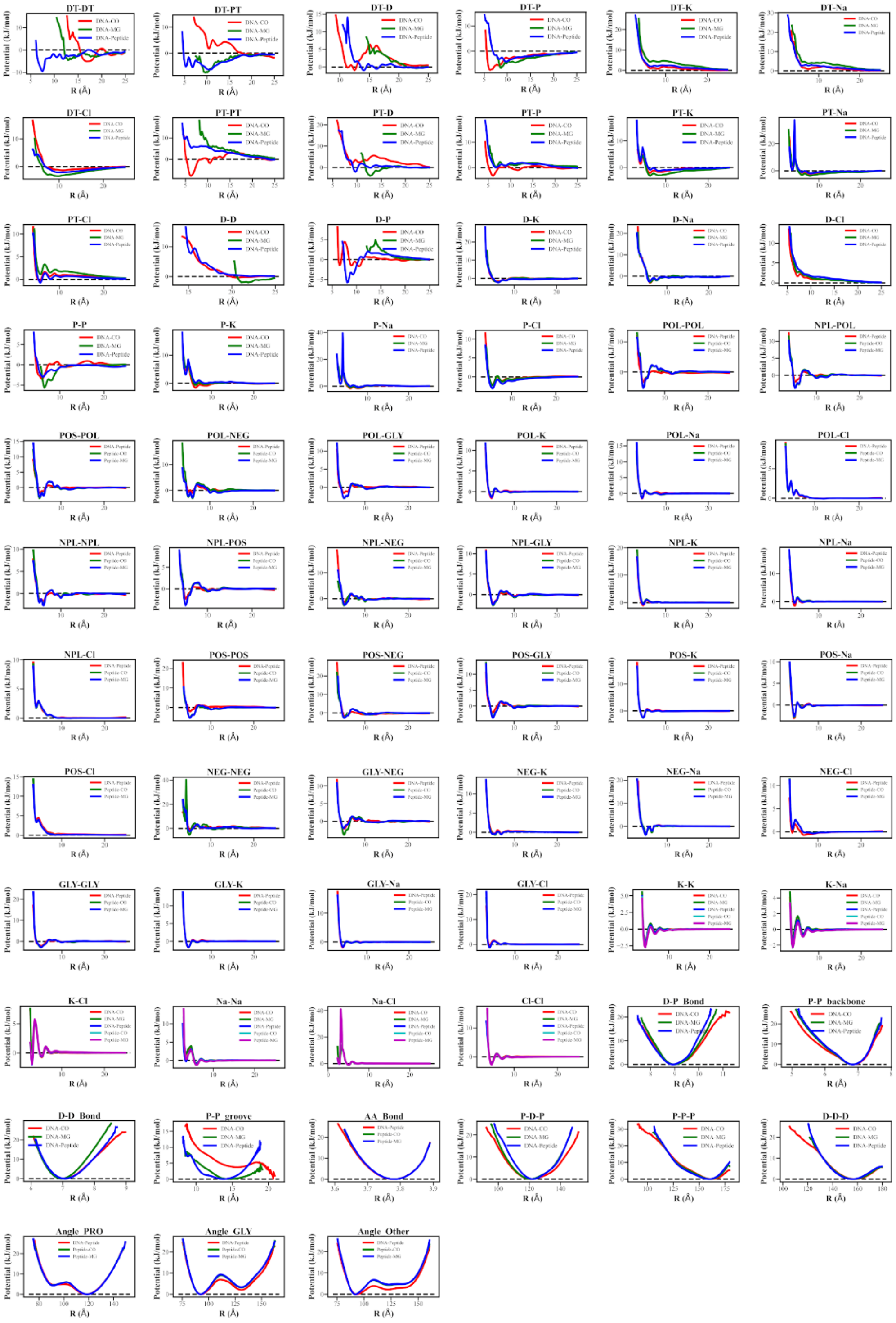

Figure S3: Comparison of the effective potentials shared in more than one all-atom MD system.

Figure S4

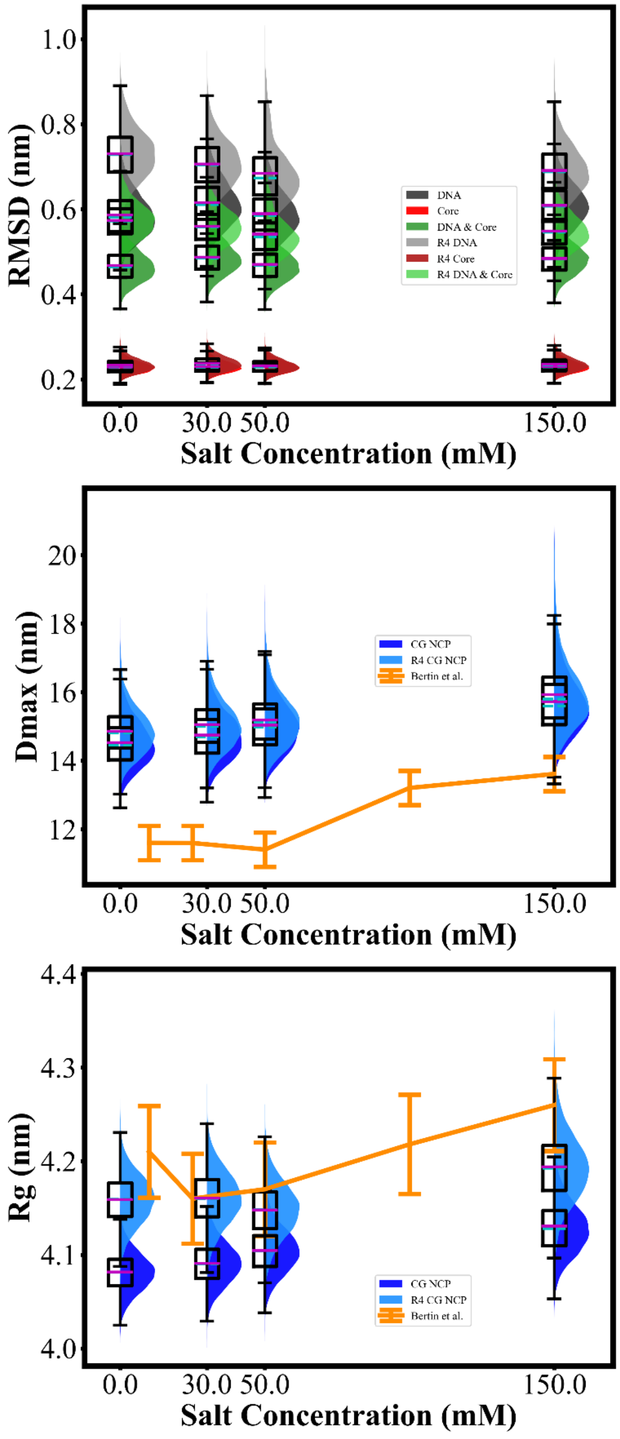

Figure S4: Comparison of the RMSD (top) D_{max} (middle), and R_g (bottom) dependence on salt concentration recorded for the two variants of the NCP model, with all DNA beads connected to the histone core (CG NCP) and with four terminal DNA beads released (R4 CG NCP). See also Figure 5 in the main text.

Figure S5

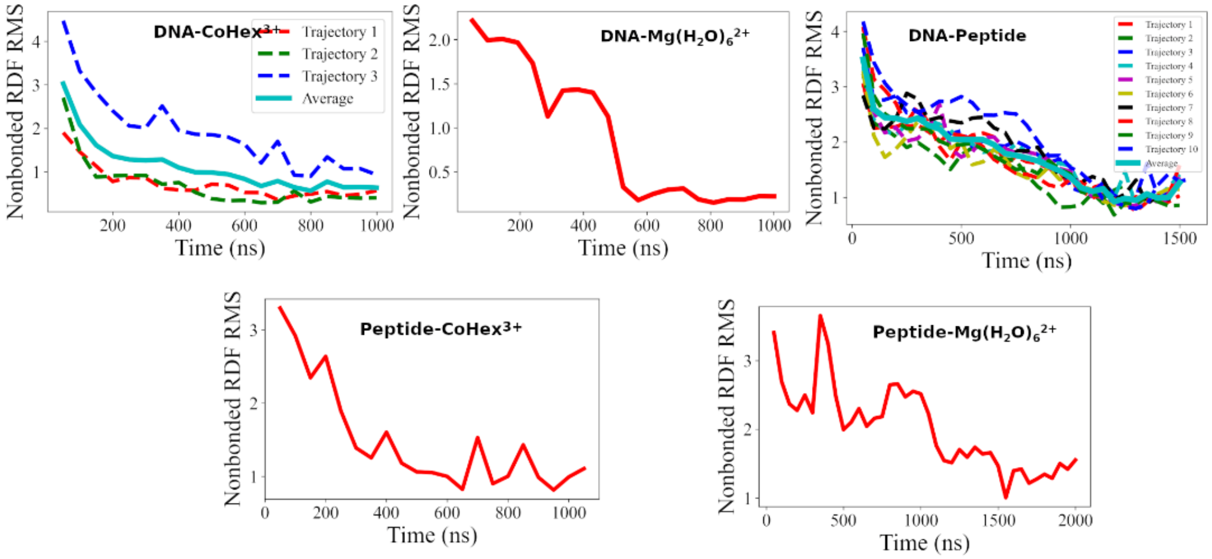

Figure S5: Root mean square fluctuation of block averaged RDF relative to the final RDF for all subsystems. The final RDF is calculated from the last 500 ns of each trajectory. Each block used for the RDF averaging is 50-ns long.

Figure S6

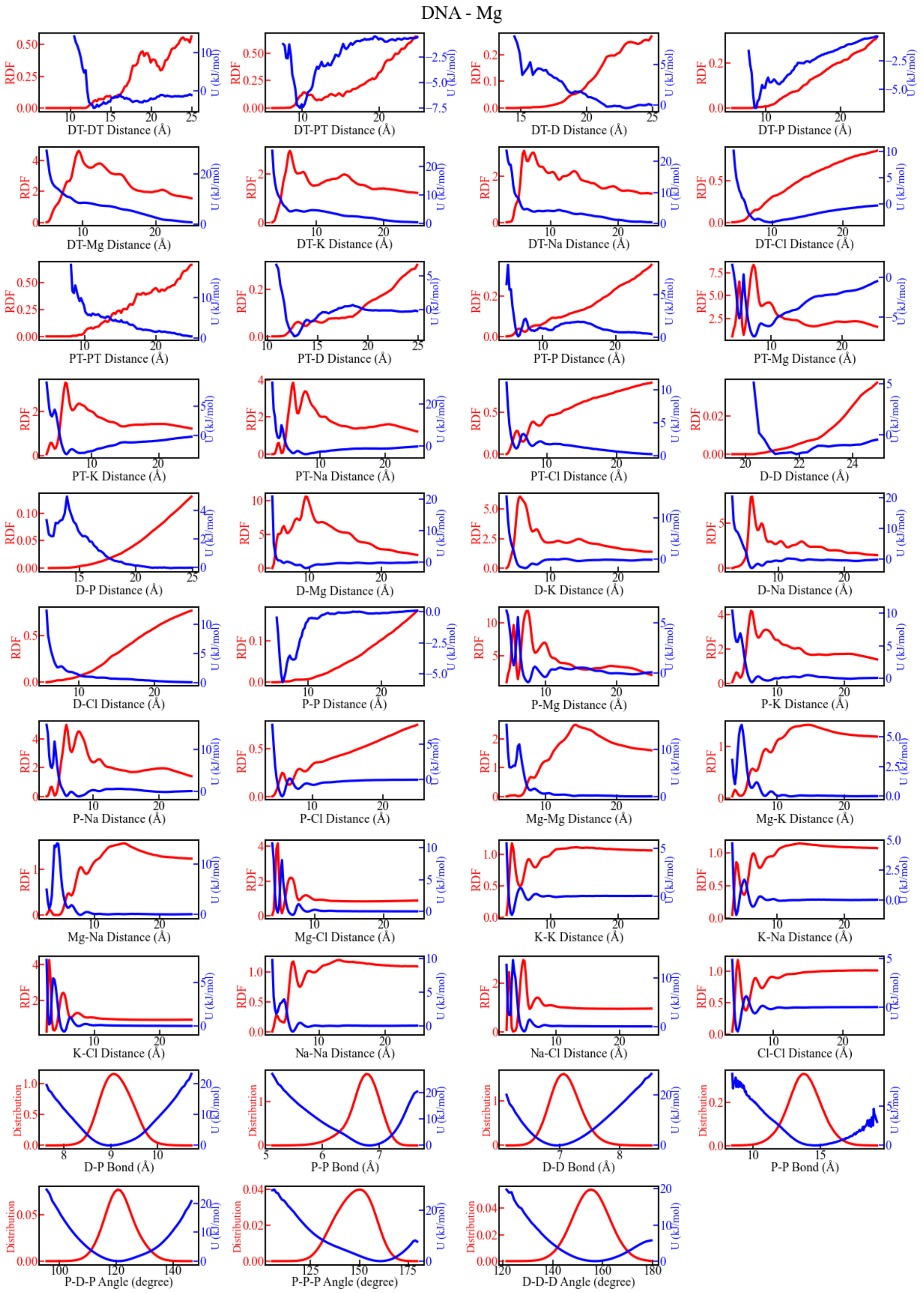

Figure S6: RDFs, angle distributions (red), and effective potentials (blue) calculated for the CG NCP particles from the all-atom MD simulations in the DNA-Mg system.

Figure S7

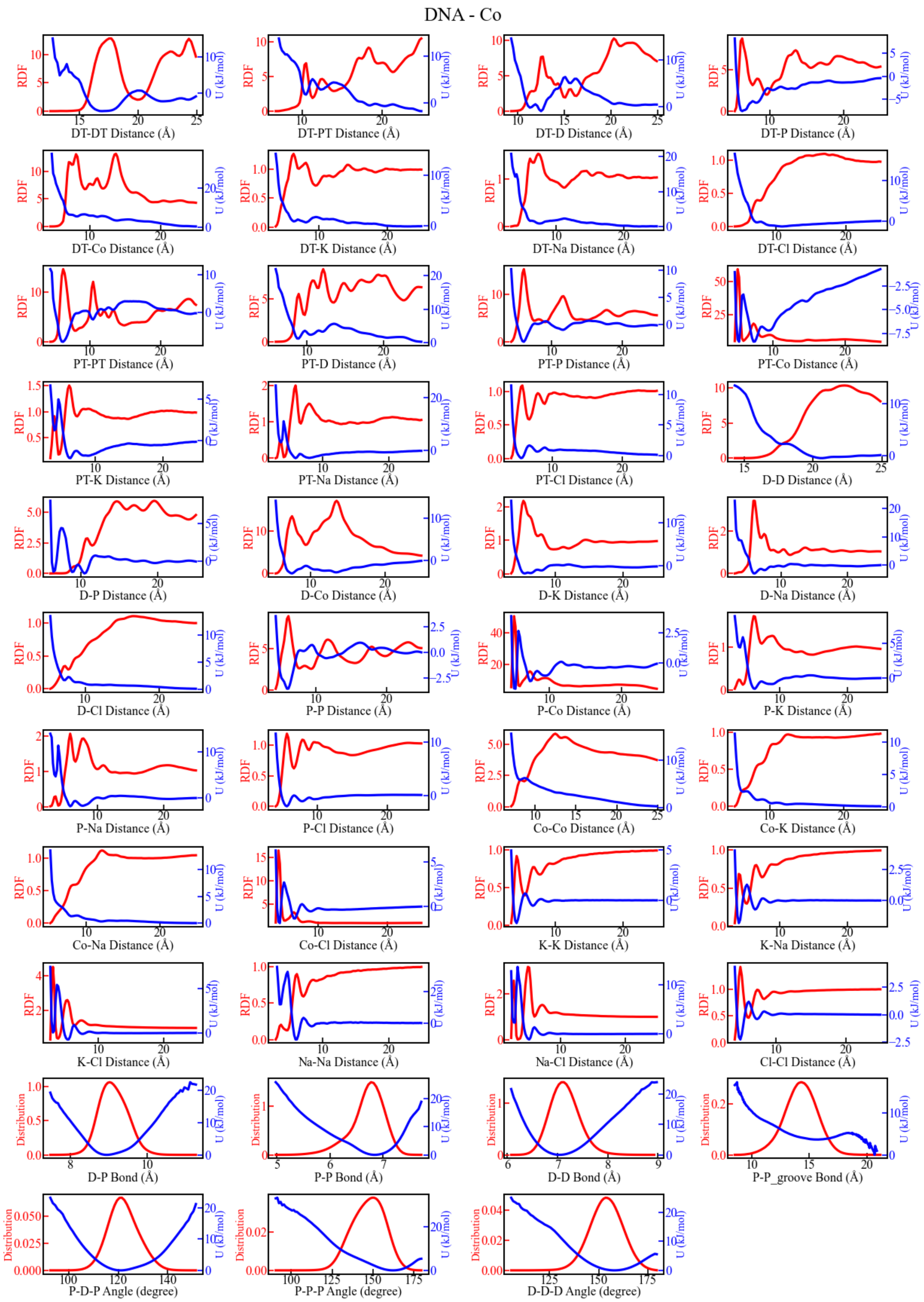

Figure S7: RDFs, angle distributions (red), and effective potentials (blue) calculated for the CG NCP particles from the all-atom MD simulations in the DNA-Co system.

Figure S8

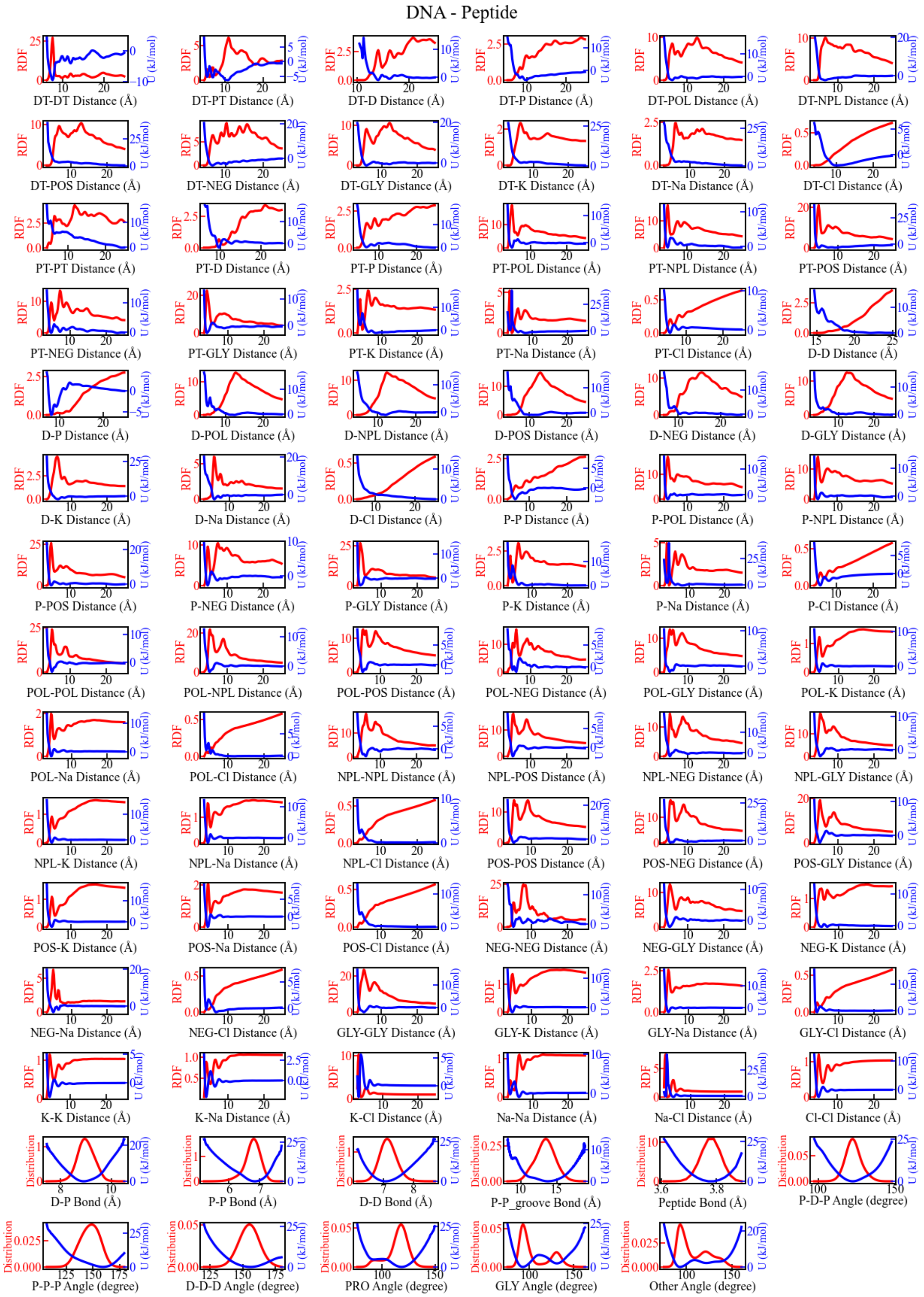

Figure S8: RDFs, angle distributions (red), and effective potentials (blue) calculated for the CG NCP particles from the all-atom MD simulations in the DNA-Peptide system.

Figure S9

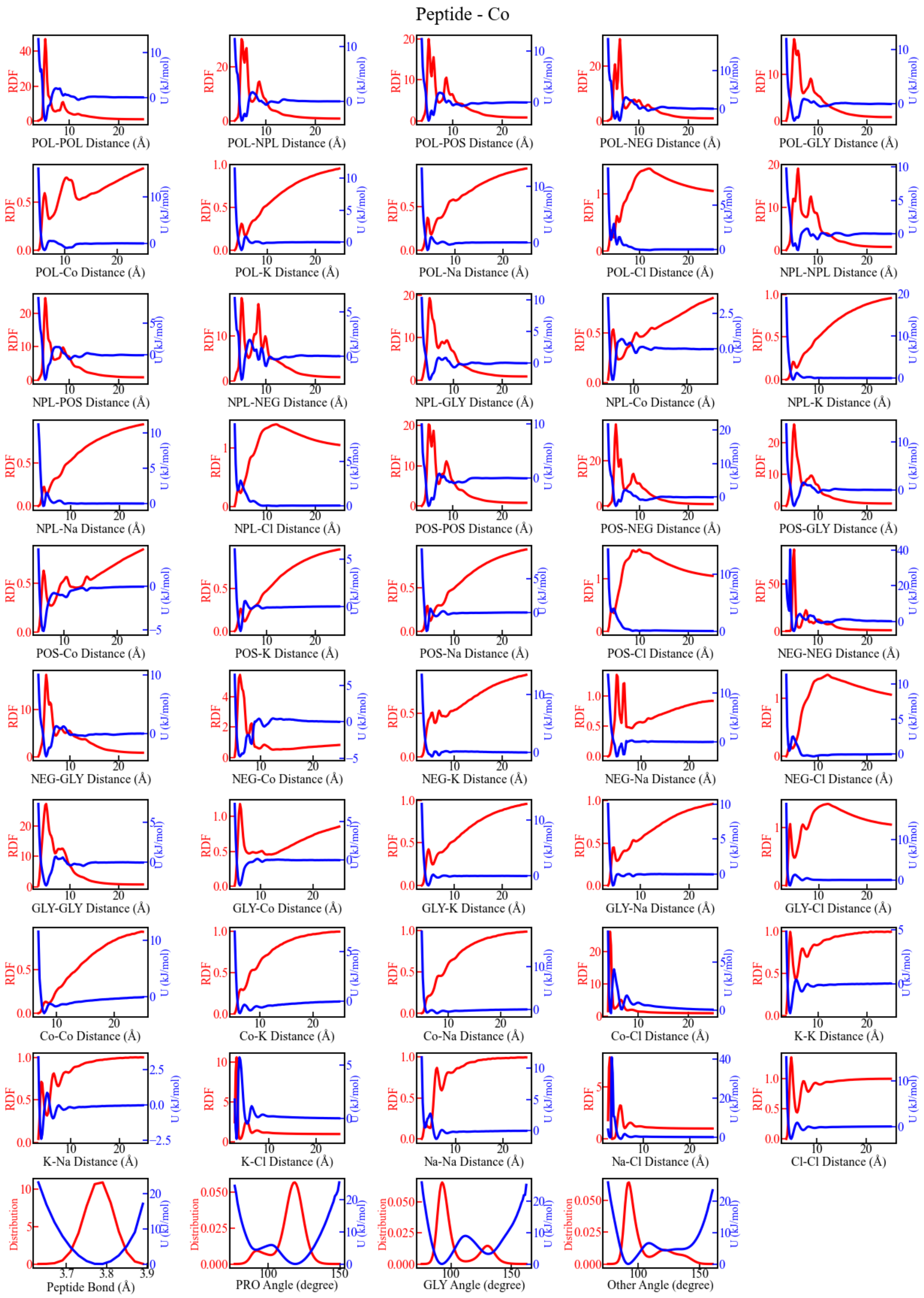

Figure S9: RDFs, angle distributions (red), and effective potentials (blue) calculated for the CG NCP particles from the all-atom MD simulations in the Peptide-Co system.

Figure S10

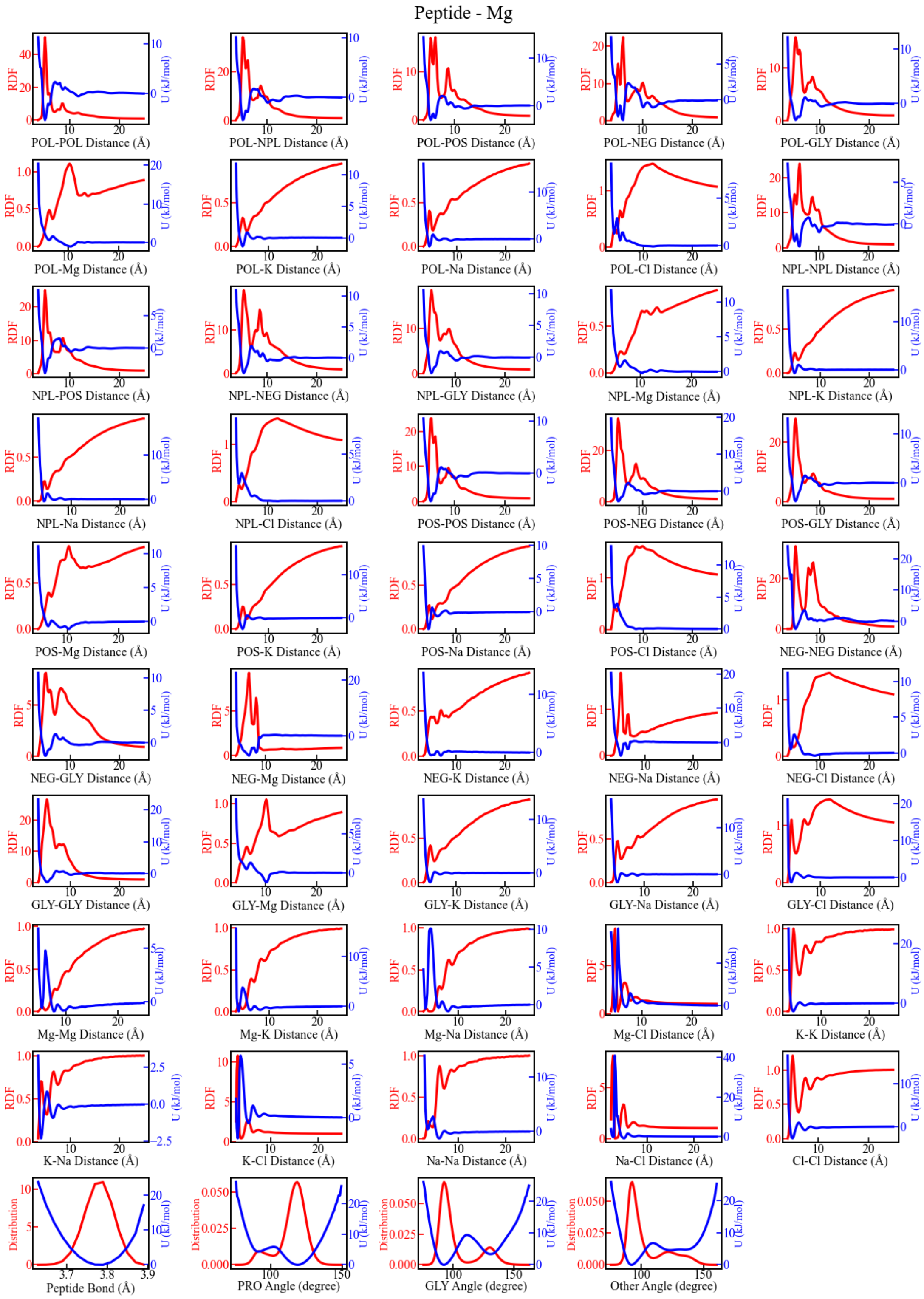

Figure S10: RDFs, angle distributions (red), and effective potentials (blue) calculated for the CG NCP particles from the all-atom MD simulations in the Peptide-Mg system.

Figure S11

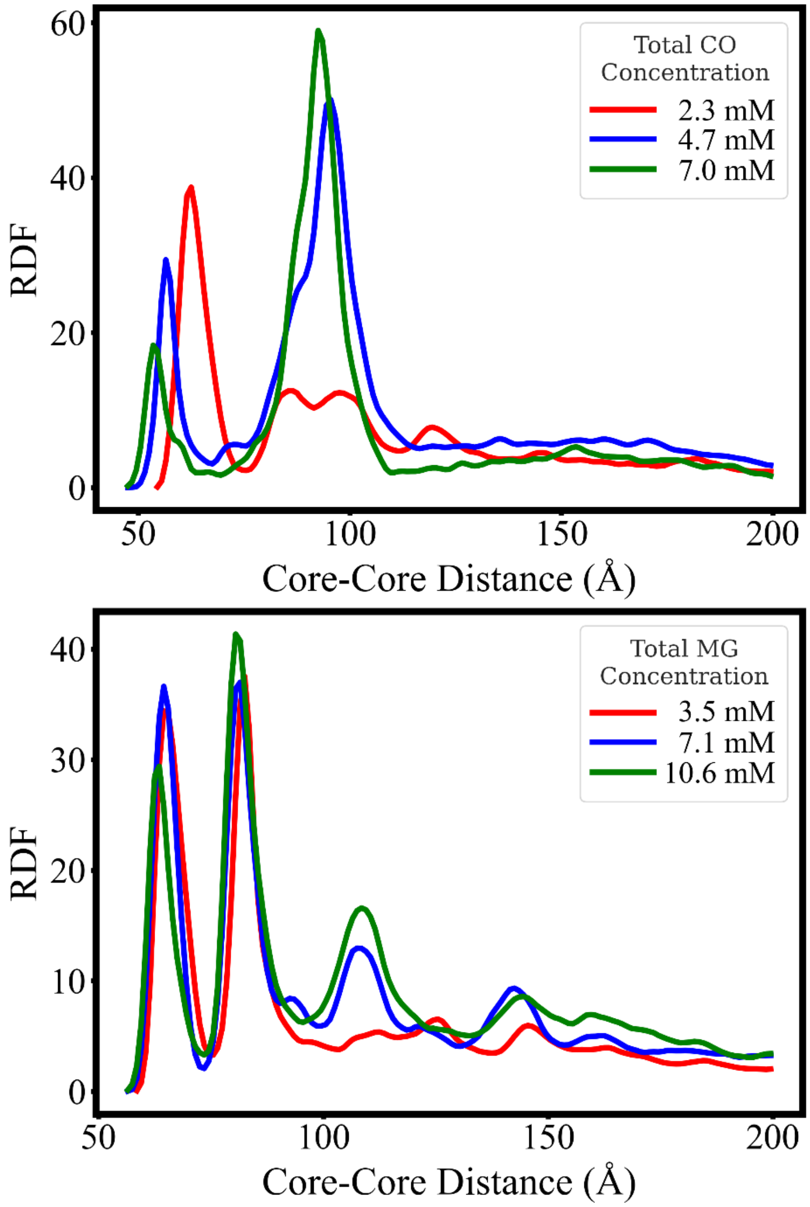

Figure S11: Core-core RDFs in dependence of different multivalent ion concentrations for the Co-20NCP (top) and Mg-20NCP (bottom) CG MD simulations.

Figure S12

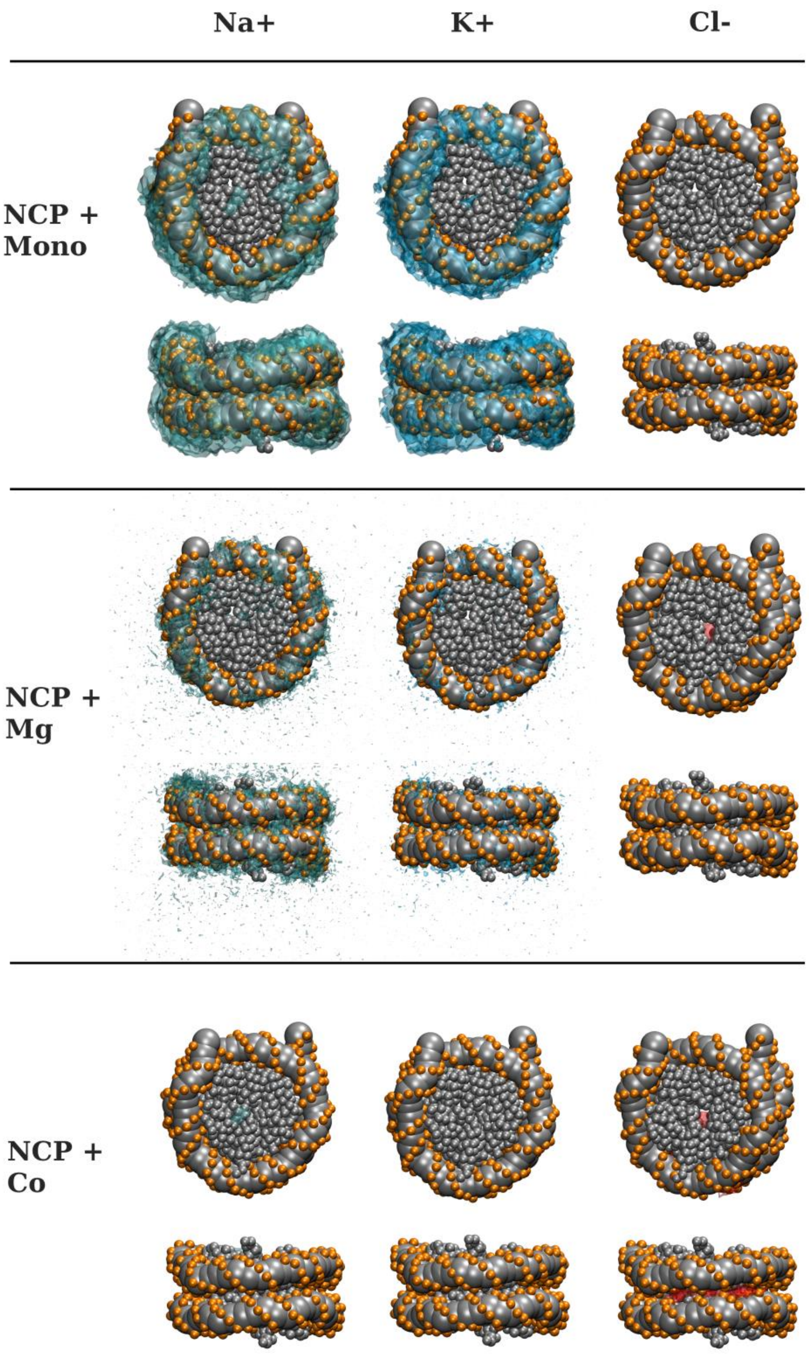

Figure S12: Comparison of monovalent ions spatial distribution function near NCP in three simulations. The presence of multivalent cations reduces the probability of finding monovalent cations near NCP. All isosurfaces are drawn with the same isovalue, representing an equal probability of finding a specific monovalent ion.
